# Evaluating clinical and neuroimaging predictors for cognitive-behavioral therapy outcome in obsessive-compulsive disorder

**DOI:** 10.1038/s41598-026-64405-y

**Published:** 2026-08-08

**Authors:** Marija Tochadse, Julia Klawohn, Christian Kaufmann, Rosa Grützmann, Anja Riesel, Stephan Heinzel, Roshan Prakash Rane, Sofia Pereira da Silva, Sarah Wellan, Sam Gijsen, Kerstin Ritter, Norbert Kathmann

**Affiliations:** 1 Department of Psychology, Humboldt-Universität zu Berlin, Berlin, Germany; 2 Hertie Institute for AI in Brain Health, Eberhard Karls Universität Tübingen, Tübingen, Germany; 3 Department of Psychiatry and Psychotherapy, Charité – Universitätsmedizin Berlin (corporate member of Freie Universität at Berlin, Humboldt-Universität zu Berlin, Berlin Institute of Health), Berlin, Germany; 4 Department of Medicine, MSB Medical School Berlin, Berlin, Germany; 5 Department of Psychology, MSB Medical School Berlin, Berlin, Germany; 6 Department of Psychology, Universität Hamburg, Hamburg, Germany; 7 Department of Educational Sciences and Psychology, TU Dortmund University, Dortmund, Germany; 8 Bernstein Center for Computational Neuroscience, Berlin, Germany; 9 Modelling of Cognitive Processes, Technical University of Berlin, Berlin, Germany

**Keywords:** Cognitive-behavioral therapy (CBT), Obsessive–compulsive disorder (OCD), Machine Learning (ML), Neuroimaging predictors, Outcome prediction, Functional connectivity, Neuroscience, Psychology, Human behaviour, Predictive markers

## Abstract

Cognitive-behavioral therapy (CBT) is the first-line treatment for obsessive-compulsive disorder (OCD), yet a significant number of patients do not achieve remission or substantial symptom relief. This study aims to enhance the prediction of CBT outcomes in OCD by integrating demographic, clinical, and neuroimaging data using machine learning (ML) models. We conduct a comprehensive analysis on a well-characterized clinical sample, employing a rigorous validation scheme to avoid data leakage, and comparing multiple ML algorithms to minimize bias. Out of four different ML models trained on demographic and clinical data, structural MRI, and resting-state MRI functional connectivity data, no model was able to predict CBT success significantly above chance level in the present sample. Although clinical and demographic data enabled 64%-66% accuracy for predicting remission, this did not reach statistical significance after permutation testing. Pre-treatment symptom severity emerged numerically as the most promising predictor of remission, aligning with previous studies, but did not pass the significance threshold in the present study. Despite efforts to identify neuroimaging predictors, neither functional nor structural MRI features significantly contributed to the prediction models. These findings suggest that robust, individualized brain-based predictions for mental health outcomes remain challenging with the available data and sample size.

**Supplementary Information:**

The online version contains supplementary material available at 10.1038/s41598-026-64405-y.

## Introduction

Cognitive-behavioral therapy (CBT) is currently the first-line treatment for internalizing disorders including obsessive-compulsive disorder^1,2^. Although it has been shown to be highly effective for OCD treatment overall^3–5^, up to half of patients do not experience remission from their disorder^5,6^ or even clinically relevant symptom relief^4,5,7,8^. This inconsistent response to treatment has motivated research into predicting which patients will benefit from psychotherapy and who is at risk of non-response (for an overview of CBT outcome studies in psychiatric disorders, see^9^).

Researchers have tried to identify OCD patients at risk of non-response based on sets of demographic and clinical variables alone^5,10–12^. Overall OCD symptom severity and individual items measuring OCD symptoms have allowed for prediction of treatment outcome^10,11^. However, predictive variables beyond symptom severity, as well as predictive performance vary considerably across studies^5,9–12^. Which clinical variables constitute robust and informative predictors for OCD treatment beyond symptom severity measures thus continues to pose an open question.

In addition to clinical features, machine learning (ML) studies have tried to boost predictive performance by including neuroimaging data as a source of information on endophenotypes. Indeed, the presence of endophenotypes in OCD is supported by some electroencephalographic (EEG) studies (e.g.^13,14^). Findings from functional magnetic resonance imaging (fMRI) suggest connectivity alterations in OCD (for a review see^15^), including aberrant connectivity in default-mode and fronto-parietal networks^16,17^. One study examined graph-theoretic measures of dynamic functional connectivity in OCD^18^. These measures can concisely characterize brain connectivity profiles and provide global summary features as a sparse set of predictors. The study demonstrated higher modularity and impaired functional flexibility in the OCD patient group.

These reports on OCD endophenotypes motivate research suggesting that resting-state fMRI may enhance ML predictive performance in OCD^19–21^. Fullana et al.^19^ controlled for baseline symptom severity and were able to predict treatment outcome based on amygdala to ventromedial prefrontal cortex connectivity alone. Their regression analyses indicated that decreased connectivity was related to better treatment outcome, suggesting a restoring effect of CBT. Reggente et al.^21^ included network connectivity, baseline symptom severity, and medication in their prediction analyses, yielding 67% balanced accuracy scores based on default-mode network connectivity. Corroborating the predictive value of resting-state fMRI, Kwak and colleagues^20^ used it to cluster OCD patients and identified patient clusters with differential outcome profiles. In addition, Grützmann and colleagues^22^ examined task fMRI data from a flanker task to predict response and remission. They found that higher sensory-motor network activity and higher initial symptom severity were related to a higher likelihood of treatment response, but no predictors were identified for remission. While these studies provide some evidence that resting-state and task-based fMRI data may contain predictive information for treatment outcome in OCD, their input features and statistical analyses vary substantially. This heterogeneity makes it difficult to identify robust predictors.

Findings on the predictive value of structural MRI features are similarly inconclusive. Some studies report prefrontal cortex volume and thickness to predict CBT outcome^23–25^; however, these studies either had very small sample sizes^24,25^ or did not employ a cross-validation scheme^23–25^. A recent large-scale study identified prefrontal cortex thickness as a CBT outcome predictor in a pediatric sample but found no evidence for structural MRI features being predictive of treatment outcome in an adult OCD patient sample^26^.

A recent meta-analysis by Vieira et al.^9^ examined research using ML to predict CBT outcome in a range of mental disorders and reports average classification accuracies of 76% for OCD. However, the authors report a clear association between classification accuracy and sample size, with larger studies showing reduced effects. Neuroimaging studies in particular often suffer from small sample sizes, raising concerns about the robustness of reported predictions. This inconsistent landscape of predictors for OCD treatment outcome, particularly the unclear role of neuroimaging data, motivates further comprehensive investigations. The present study aims to systematically evaluate the predictive power of demographic, clinical, and both structural and functional neuroimaging data. We compute unimodal ML models first and subsequently combine data from multiple modalities to investigate multimodal analysis advantages.

In this study, we aim to assess CBT outcome prediction in OCD using a combination of demographic, clinical, and brain measures. We employ a relatively large dataset with a clinically characterized sample regarding OCD symptomatology and unspecific clinical features (analysis of flanker task fMRI data of a part of this sample was published in^22^). Methodologically, strict analysis and validation standards were adopted. A standardized ML-pipeline allowed us to compare multiple ML-models to minimize model-related bias. A repeated nested cross-validation scheme was used for parameter selection, and permutation testing was applied to evaluate prediction accuracy significance.

## Methods

### Participants

The dataset comprised a consecutive patient sample collected at the OCD outpatient clinic at Humboldt-Universität zu Berlin. Patients were diagnosed with OCD by trained clinicians based on the German version of the Structured Clinical Interview for DSM-IV^27^ and had a severity score of ≥ 12 in the Yale-Brown Obsessive-Compulsive Scale (Y-BOCS^28^). Patients with OCD (further inclusion criteria were: no prominent suicidal ideation, no lifetime substance dependence, no borderline personality disorder, no comorbid psychotic disorders, no history of head trauma or neurological diseases) received CBT within the standard outpatient framework of up to 80 sessions after being screened for participation in the study. The number of sessions actually attended ranged from 8 to 98 (mean = 47.0, SD = 19.8). Values exceeding 80 sessions reflect approved session extensions consistent with routine clinical practice in the German statutory health insurance system.

The study was approved by the ethical review board of the Humboldt-Universität zu Berlin. All data collection adhered to institutional ethical requirements and the 1964 Helsinki Declaration and its amendments. All patients gave written informed consent after receiving verbal and written explanation of the purpose and procedures of the study.

**Fig. 1:**
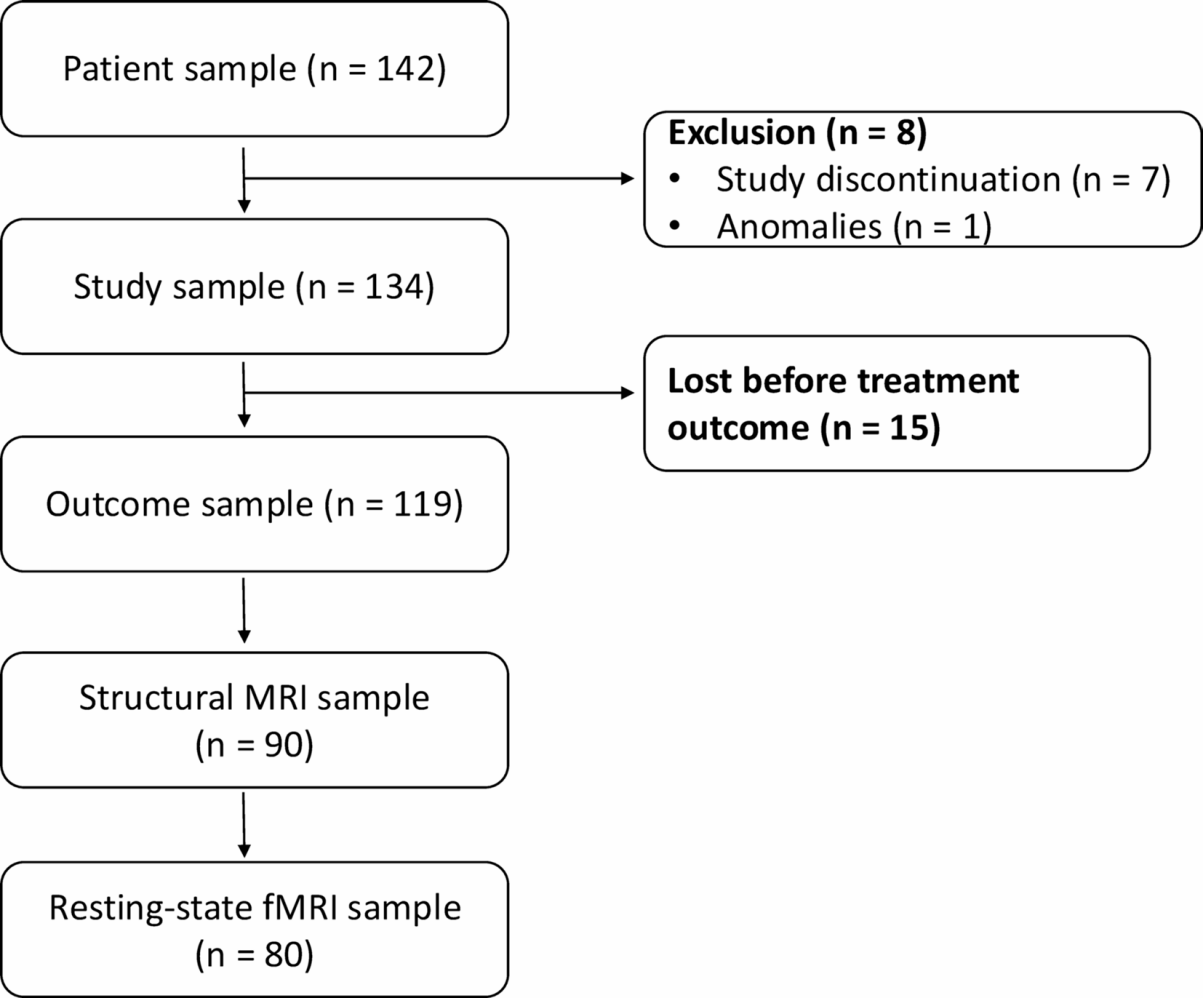
Sample size and participant inclusion based on data availability and label information.

A participant flow chart is provided in Fig. 1. The initial sample consisted of 142 patients. Seven patients were excluded from the study due to withdrawal from the study and one patient due to neurological anomalies. Another 15 patients were missing treatment outcome information and were therefore excluded from the prediction analysis, leaving 119 patients in the tabular data analysis sample. Out of 119 patients, 72 had a significant treatment response and 60 showed remission. Structural MRI scans were available for 90 patients, with a subsample of 80 patients also obtaining a resting-state fMRI scan. Maximum sample size available per modality was used when conducting the ML analyses.

### Demographic and clinical data

Demographic and clinical variables spanned 31 features. Clinical scales related to OCD included the six subscales (washing, obsessing, ordering, checking, neutralizing, and hoarding) of the Obsessive-Compulsive Inventory-Revised (OCI-R^29^), as well as two trait-like motivational dimensions of OCD (harm avoidance and incompleteness), assessed with the Obsessive-Compulsive Trait Core Dimensions Questionnaire (OCTCDQ^30^), and the age at first OCD diagnosis. Baseline measures of OCD severity (Y-BOCS subscale sum scores for compulsions and obsessions) were also included. Clinical scales not directly related to OCD included severity of depressive symptoms, trait anxiety, dimensional scores of personality disorders (SCID-II^27^), Temperament and Character Inventory sum scores (TCI^31^), and executive functioning scores from the Kölner ADHS-Test für Erwachsene (KATE^32^). Finally, comorbid depression and medication status and type were considered in the analyses. Table 1 gives an overview of the demographic and clinical input features.

The number of CBT sessions received was not included as a predictor, as it is unavailable at the pre-treatment decision point targeted by the model. Including a post-treatment variable would constitute temporal data leakage and compromise the prospective utility of the pipeline. As a supplementary analysis, session count was used as the sole predictor and as an additional predictor alongside all clinical features to confirm that treatment duration does not carry unique predictive variance beyond pre-treatment characteristics (balanced accuracy: 50%–55% as sole predictor for response and 47%−48% when combined with the full clinical feature set; 48%–53% as sole predictor for remission and 64%−66% when combined with the full clinical feature set, exactly mirroring clinical feature prediction values).

Twenty-six patients discontinued CBT before completing the planned treatment protocol. To maximize the available sample size, treatment outcome for these patients was determined using a last-observation-carried-forward approach based on their final available Y-BOCS assessment. Early discontinuation was not assumed to be equivalent to treatment failure, as 8 of these patients met the response criterion and 6 met the remission criterion. Furthermore, the number of completed treatment sessions varied substantially within this subgroup (mean = 29 sessions) and was comparable between responders and non-responders as well as between remitters and non-remitters. These patterns indicate that their clinical categorizations reflect genuine treatment trajectories rather than mere premature termination, and support the inclusion of these participants in the analyses.

**Table 1:** Mean values (SD) for demographics and clinical variables separately for patients showing response or non-response and remission or non-remission. For sex, percent female is shown, and for the binary variables medication status and comorbid depression the percentage of patients with medication and comorbid depression, respectively, are shown. Y-BOCS: Yale-Brown Obsessive Compulsive Scale; OCTCDQ: Obsessive-Compulsive Trait Core Dimensions Questionnaire; OCI-R: Obsessive-Compulsive Inventory-Revised; MADRS: Montgomery-Asberg Depression Rating Scale; BDI-II: Beck Depression Inventory-II; STAI-TRAIT: State-Trait Anxiety Inventory; WST: Wortschatztest [German vocabulary test]; NEOFFI: NEO Five Factor Inventory; KATE: Kölner ADHS-Test für Erwachsene [Cologne ADHD test for adults]; SCID-II: Structured Clinical Interview for DSM-IV Personality Disorders; TCI: Temperament and Character Inventory. Unpaired *t*-tests were computed for all dimensional variables for the levels of response and remission and Bonferroni corrected for multiple comparisons, * denotes *p* < 0.05, ** denotes *p* < 0.01.

	Response	Non-response	Remission	Non-remission
Sex (% female)	46%	62%	43%	61%
Age	33 (10)	31 (9)	32 (9)	32 (10)
Medication status (% medicated)	46%	40%	42%	46%
Comorbid depression (% comorbid)	57%	55%	50%	63%
Y-BOCS obsessions scale	11 (4)	11 (3)	10 (4)	13 (2) **
Y-BOCS compulsions scale	12 (3)	11 (4)	11 (4)	13 (3) *
OCTCDQ harm avoidance scale	10 (4)	10 (3)	9 (4)	11 (3)
OCTCDQ incompleteness scale	15 (6)	15 (5)	13 (6)	17 (4) *
OCD onset (DSM diagnosis)	24 (10)	23 (8)	24 (10)	23 (9)
OCI-R washing scale	4 (4)	5 (4)	3 (4)	6 (4) **
OCI-R checking scale	7 (4)	6 (3)	6 (4)	6 (3)
OCI-R ordering scale	4 (4)	5 (4)	4 (3)	5 (4)
OCI-R obsessing scale	7 (4)	7 (3)	7 (4)	7 (3)
OCI-R hoarding scale	2 (3)	2 (3)	2 (2)	3 (3)
OCI-R neutralizing scale	3 (3)	3 (3)	3 (3)	3 (3)
MADRS	13 (9)	14 (9)	12 (9)	16 (9)
BDI-II	18 (11)	20 (11)	16 (10)	23 (11) *
STAI-TRAIT	52 (12)	53 (8)	49 (11)	57 (8) **
WST	31 (4)	31 (4)	32 (4)	30 (4)
NEOFFI neuroticism scale	28 (9)	29 (7)	26 (9)	31 (7) **
NEOFFI extraversion scale	23 (7)	23 (7)	24 (7)	22 (7)
KATE emotionality scale	5 (3)	5 (3)	4 (3)	6 (3)
KATE executive functions scale	11 (7)	11 (8)	9 (7)	13 (7) *
SCID-II avoidant scale	3 (2)	3 (2)	2 (2)	3 (2)
SCID-II obsessive-compulsive scale	5 (2)	4 (2)	4 (2)	5 (2)
SCID-II schizotypal scale	2 (1)	2 (2)	2 (1)	2 (2)
SCID-II borderline scale	4 (3)	4 (3)	4 (2)	5 (3)
TCI anticipatory worry scale	6 (3)	6 (2)	5 (3)	7 (2) *
TCI fear of uncertainty scale	5 (2)	6 (1)	5 (2)	6 (1)
TCI shyness scale	4 (2)	5 (2)	4 (2)	5 (2) **
TCI fatigability scale	6 (2)	6 (2)	5 (2)	6 (2)

### Neuroimaging data (acquisition & preprocessing)

MRI data was acquired with a 3 T Siemens TrioTim scanner (Siemens Corp.) at the Berlin Center for Advanced Neuroimaging with a 32-channel coil. For the structural MRI scan the MPRAGE sequence was used. Acquisition parameters were as follows: slice thickness: 0.91 mm, echo time: 0.00481 s, repetition time: 2.44 s, inversion time: 1.1 s, flip angle: 8, number of slices: 192, field of view: 234 mm, matrix size: 256 × 256. For resting-state fMRI an echo planar imaging sequence was used and field maps in anterior-posterior and posterior-anterior direction were acquired prior to the resting-state scan. The following parameters were used: slice thickness: 2 mm, echo time: 0.0348 s, repetition time 1.05 s, flip angle: 65, number of slices: 72, matrix size 104 × 104, volumes: 350.

For preprocessing, we used the HALFpipe pipeline^33^, an fMRI preprocessing tool built on top of fMRIPrep^34^ that includes additional preprocessing features. Structural MRI preprocessing was done with FreeSurfer’s^35^ recon-all function inside HALFpipe. The Brainnetome atlas^36^ was used for fMRI and structural MRI parcellation. For fMRI data, we applied grand mean scaling and temporal filtering (0.01–0.1 Hz) and performed motion artifact removal with ICA-AROMA^37^. No smoothing or slice-time correction were applied. Quality control was done by visual inspection of HALFpipe’s quality assessment output, including T1 skull-stripping, spatial normalization, signal-to-noise ratio in the EPI sequence data, motion-related confounds, ICA components, and EPI spatial normalization.

We extracted 496 structural MRI features, including 216 cortical volumes, 214 cortical thickness features, and 66 subcortical volumes. For resting-state fMRI, we extracted correlation matrices between 263 cortical and subcortical regions from the Brainnetome atlas. Regions that had missing values in the correlation matrix from at least one participant were excluded, resulting in 248 regions. For each patient, the correlation matrix was vectorized and used as input features in the ML analysis. To reduce the dimensionality of the functional connectivity feature space, principal component analysis (PCA) was applied within the training folds of the outer cross-validation scheme; the number of retained components was treated as a hyperparameter and optimized through the inner cross-validation loop, with candidate values of (10, 20, 30, 40, 50). To ensure our predictive outcomes were not strictly constrained by our choice of dimensionality reduction, Independent Component Analysis (ICA) was included as a sensitivity analysis. This allowed us to explicitly test whether isolating statistically independent sources within the connectivity data could improve prediction compared to the orthogonal, variance-maximizing components generated by PCA. We used a fixed number of components set to the modal optimum identified across all outer folds and model configurations in the PCA analyses (30 components for response prediction explaining 76% of variance, and 20 components for remission prediction explaining 66% of variance). A fixed rather than cross-validated component count was used because the iterative convergence algorithm for FastICA in scikit-learn is computationally more expensive than PCA’s closed-form decomposition, making a full grid search prohibitive.

Additionally, global graph features were extracted from the correlation matrices and comprised weighted implementations of transitivity, global efficiency, clustering coefficient, triangle number, density, and small-world coefficients sigma and omega. Graph characterization was deliberately restricted to nine global network measures as a principled strategy to summarize whole-brain network organization in a compact, interpretable feature space commensurate with the available sample size. Node-level graph metrics were not included because, applied comprehensively across the parcellation, they would defeat the purpose of a compact network summary. A targeted supplementary analysis restricting input features exclusively to amygdala-vmPFC functional connectivity (Fullana et al., 2017) was additionally conducted and is detailed in the Supplementary Material.

### Outcome measures

Two CBT outcome measures were based on the Y-BOCS score at the end of therapy. The Y-BOCS measures the severity of impairment resulting from the patients’ obsessions and compulsions constituting a major pillar in the clinical assessment. Remission was defined as a Y-BOCS value smaller than 12 at the end of treatment (see^5,38^). Response was defined as at least 35% reduction in Y-BOCS from baseline to end of therapy^5^. The last measurement available for an individual participant was counted as the post-treatment assessment (intent-to-treat analysis).

### Analysis

**Fig. 2:**
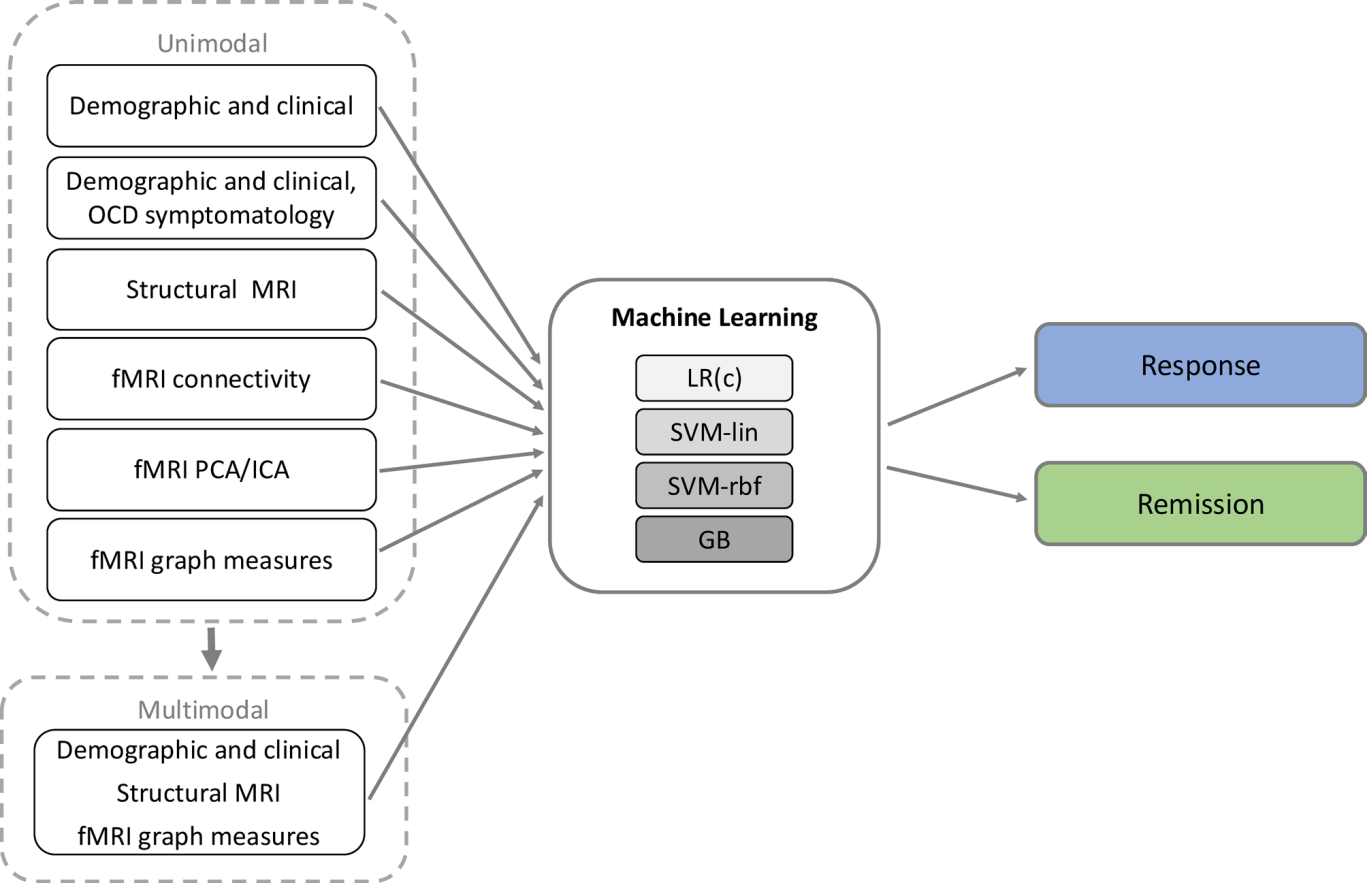
Schematic representation of the analysis outline. Unimodal and multimodal input features were used to predict ‘Response’ and ‘Remission’ with four ML models. LR(c): logistic regression, SVM-lin: support vector machine with linear kernel, SVM-rbf: support vector machine with radial basis function, GB: gradient boosting.

Analyses were computed using an extended version of the lab’s ML pipeline reported in Rane et al.^39^. For an overview of input modalities and outcomes see Fig. 2. We included demographic and clinical variables, structural MRI features, resting-state fMRI features independently, and multimodal analyses with demographic and clinical variables combined with structural MRI and resting-state fMRI in the prediction analyses. One model included demographic data and clinical variables that were unspecific to OCD to investigate clinical predictors independent of baseline symptom severity effects. In a second model, we included all clinical features in order to exploit all available clinical information to maximize outcome prediction. For multimodal analyses, the demographic and clinical data including OCD-specific variables were concatenated with structural MRI data and fMRI graph features.

Each feature was standardized to have zero mean and unit variance across all subjects (mean and variance were estimated only on the training data and then applied to the test data). The ML pipeline comprised a two-stage cross-validation (CV) scheme with 7 outer folds and an inner 5-fold CV procedure; all splits were stratified by outcome label to preserve class proportions across folds. The pipeline was repeated 10 times with different random seeds for splitting training and test data to account for variation in performance due to the reduced sample sizes. Missing values were imputed within each outer CV fold: the median (continuous variables) and mode (categorical variables) were computed from the training fold only and subsequently applied to impute missing values in both the training and held-out test fold, avoiding data leakage. Two linear and two non-linear ML models were evaluated, comprising logistic regression (LR), linear support vector machine (SVM-lin^40^), and kernel SVM with a radial basis function (SVM-rbf^41^) and a gradient boosting (GB^42^) as the non-linear models. For SVM-lin we used the liblinear^43^ implementation and as the ensemble method GB we used XGBoost^44^. Hyperparameters were tuned in the inner CV folds and are listed in Supplementary Table 1. In addition to the primary binary classification tasks, we conducted a supplementary regression analysis predicting percent Y-BOCS reduction as a continuous outcome to ensure our results were not artifacts of threshold-dependent outcome operationalization (detailed in Supplementary Material).

We evaluated model performance with the balanced accuracy metric, which averages accuracy over both outcome classes and thus considers both sensitivity and specificity. Performance measures were averaged over 7 outer folds and 10 repeated runs, i.e. 70 runs per model. Additionally, we performed permutation testing to determine if classifier performance was significantly above chance level^45^. Statistical significance was assessed using a fold-level permutation framework, as our aim was to evaluate whether models demonstrate robust generalization across independent held-out samples. Specifically, training labels were randomly shuffled, and model performance was evaluated on the corresponding non-shuffled test set data. This procedure was repeated 1000 times to generate a null distribution from which p-values were calculated. We further evaluated the statistical power of this approach relative to a pipeline-level permutation framework in a simulation-based sensitivity analysis (Supplementary Analysis 3).

### Confounds

First, we tested whether the common confounders sex and age in studies employing neuroimaging-derived predictors confounded the analyses in our dataset. For this, we predicted response and remission from sex and age separately. Prediction of both response and remission was at chance level for the predictor age; therefore, we did not treat it as a confounder in following analyses. For sex, prediction of treatment response was at chance level, and remission was predicted with 59% balanced accuracy, yet not passing the significance threshold. To rule out any confounding effects nonetheless, we opted to eliminate the influence of sex on remission prediction by oversampling^46^. Oversampling was applied strictly within the training fold of each outer cross-validation iteration: we sampled with replacement from male subjects until the sex distribution was similar across remitted and non-remitted patients in the training data only. The held-out test fold was never resampled and always contained the original, unmodified subjects, ensuring no leakage from the oversampling procedure. Sex was not included as a predictive variable in either ML analysis. In contrast, comorbidity and medication status were retained as predictive features because they represent clinical characteristics that may carry genuine prognostic information for treatment response. Post-hoc checks confirmed no significant difference in medication or comorbidity status between response and remission groups, ensuring this methodological choice did not bias the overall null results.

### Feature importance

To interpret model predictions and assess the contribution of individual features, we computed SHapley Additive exPlanations (SHAP^47,48^) values for all four model types. SHAP is a game-theoretic framework that attributes the output of a model to its input features by computing each feature’s marginal contribution across all possible subsets of features. This yields a signed importance value per feature per sample, indicating both the magnitude and direction of each feature’s influence on the predicted outcome for that individual. For tree-based models (gradient boosting), exact SHAP values were computed using the tree structure. For linear models (logistic regression, linear SVM), SHAP values were computed under an independence assumption between features. For the kernel SVM with radial basis function (SVM-RBF), which has no closed-form SHAP solution, a model-agnostic kernel-based approximation was used: SHAP values were estimated by fitting a locally weighted linear model around each prediction, using a sample of the training data as the background reference distribution for the integration step.

SHAP values were computed separately for each outer cross-validation fold, applied to the held-out test samples using the model fitted on the corresponding training fold. This ensured that importance values were derived from truly out-of-sample predictions rather than in-sample fits. The resulting per-sample SHAP values were aggregated across all folds and pipeline repetitions to obtain dataset-level importance estimates. Feature importance was summarized as the mean absolute SHAP value across samples, folds, and repetitions, providing a stable, aggregate measure of each feature’s contribution. Computing SHAP values across all folds and repetitions additionally allowed us to assess the stability of feature importance estimates. Features whose SHAP values were consistent across folds were considered robustly informative, whereas features with high cross-fold variance were treated as unstable and interpreted with caution.

## Results

### Demographics

The majority of patients (full sample *n* = 119, 62 female) were labeled as responders (60.5%, 72 patients), while almost equal numbers of patients were remitters (50.4%, 60 patients) and non-remitters (49.6%, 59 patients). Nineteen (16.0%) patients achieved treatment response but not remission, and seven (5.9%) remitted patients were not counted as responders. A contingency table showing response and remission outcome numbers is presented in Table 2.

An overview of sociodemographic and clinical characteristics broken down by response and remission status is presented in Table 1. More males than females were responders and remitters (64% and 66%), however the sex distribution between outcome groups did not differ significantly. Significance testing was performed with unpaired t-tests for continuous and chi-squared tests for categorical variables and Bonferroni corrected for multiple comparisons. None of the clinical and demographic variables differed significantly between responders and non-responders. Remitters and non-remitters showed significant differences in several OCD-related and OCD-unspecific clinical scores (indicated with asterisks in Table 1), notably in Y-BOCS sub-scores with non-remitting patients exhibiting greater baseline Y-BOCS scores.

**Table 2:** Contingency table presenting participant numbers across two outcome labels ‘Response’ and ‘Remission’.

	Non-Remission	Remission	All
Non-Response	40	7	47
Response	19	53	72
All	59	60	119

### ML predictions

**Fig. 3:**
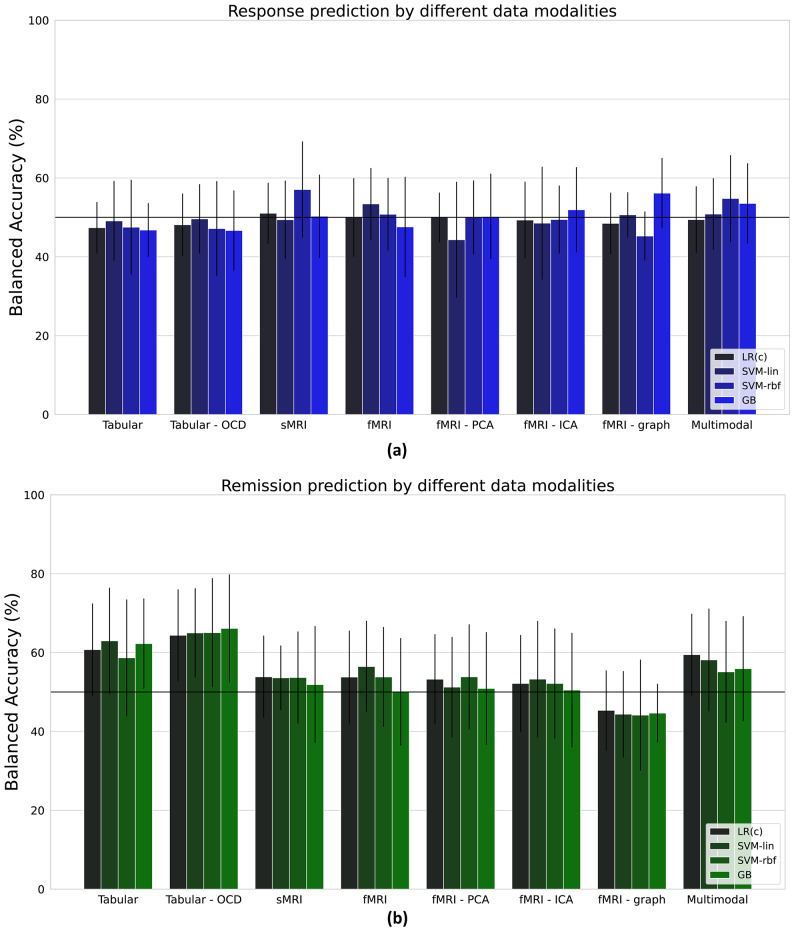
(**a**) Balanced accuracies for response prediction from unimodal and multimodal input data. (**b**) Balanced accuracies for remission prediction from unimodal and multimodal input data. Error bars show standard deviation across all outer validation folds and repeated runs and the horizontal line depicts chance level prediction. ’Tabular’ denotes demographic and OCD-unspecific clinical variables, ’Tabular - OCD’ refers to demographic and clinical data including all OCD-specific variables. sMRI: structural MRI features, fMRI: resting-state fMRI functional connectivity features, fMRI – PCA: principal components obtained from PCA on resting-state fMRI functional connectivity features, optimized as hyperparameter across [10, 20, 30, 40, 50] candidate values, optimal number of components = 30/20 for response and remission, respectively, fMRI – ICA: modal optimum of 30/20 components for response/remission, obtained from ICA on resting-state fMRI functional connectivity features, fMRI – graph: global graph measures based on resting-state fMRI functional connectivity, Multimodal: concatenation of ’Tabular’ (including OCD-specific variables), ’sMRI’, and ‘fMRI – graph’ input features.

The results of the ML predictions for response and remission outcomes are shown in Fig. 3. The unimodal prediction analyses for treatment response based on demographic and clinical data, structural MRI, and resting-state fMRI data did not perform above chance level (balanced accuracy ranging from 44% to 57%). The best-performing model was an SVM-rbf classifier trained on structural MRI data with 57% balanced accuracy but did not reach significance in the permutation test. For resting-state functional connectivity, the number of PCA components most frequently selected in the inner cross-validation was 30; therefore, ICA was performed using 30 components and yielded consistent chance-level results. The multimodal response prediction also performed at chance level with 49%–55% balanced accuracy for different model types.

**Fig. 4:**
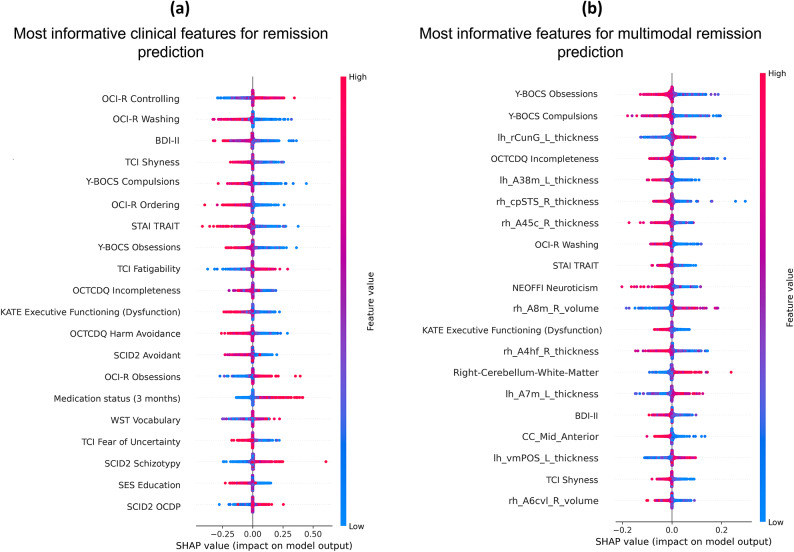
(**a**) Most informative Tabular clinical features (including OCD variables) for remission prediction. (**b**) Most informative features for multimodal remission prediction. SHAP summary plot of the top 20 features integrating clinical, structural MRI, and graph-based data to predict remission. Features are ranked by mean absolute SHAP value. The x-axis indicates the magnitude and direction of a feature’s effect on the model’s prediction (positive values favor remission). Dot color represents the magnitude of the underlying feature for an individual patient.

For remission prediction, neuroimaging-based ML analyses performed at chance level (44%–56%). For resting-state functional connectivity, the most frequently selected number of PCA components was 20. ICA using the same number of components yielded comparable chance-level results, indicating that the choice of dimensionality reduction approach did not meaningfully influence prediction performance. A targeted supplementary analysis utilizing solely amygdala-vmPFC connectivity features similarly yielded chance-level predictions for both response and remission (balanced accuracies 46–51%; see Supplementary Material). Demographic and clinical information predicted remission with 59%–63% when excluding OCD-specific clinical information, and 64%–66% with all clinical predictors (mainly based on severity of OCD symptoms at baseline). However, none of the models passed the significance level. Inclusion of neuroimaging information did not raise prediction accuracies above the significance level. Supplementary regression analyses predicting continuous percent symptom change from all clinical features yielded similarly null results (R² < 0 across all models), aligning with the classification findings (see Supplementary Material).

### Feature importance

SHAP feature importance was computed for all models and is reported descriptively, given that overall prediction did not reach significance. It is crucial to note that feature importance rankings derived from models performing at chance level must be interpreted with caution. These rankings are provided for descriptive transparency only and should not be interpreted as evidence of clinically meaningful predictors. Furthermore, SHAP values for the SVM-RBF model were estimated using a kernel-based approximation, which may introduce additional uncertainty compared with the exact SHAP calculations available for linear or tree-based models. Figure 4a and b show the results for the clinical and multimodal remission prediction models, which were of primary interest given their prediction accuracies. All SHAP figures for response prediction are provided in the supplement, along with the figures for remission prediction models based on structural MRI, graph-based features, and clinical data (excluding OCD variables).

For the clinical predictor models, the features assigned the highest importance overlapped substantially with variables showing significant group differences in Table 1. For response prediction, OCI-R checking severity, TCI fatigability, and socioeconomic status (education) received the highest mean absolute SHAP values, followed by depression severity (BDI-II), trait anxiety (STAI-TRAIT), and OCTCDQ incompleteness (Supplementary Fig. 5). For remission prediction, OCI-R checking and washing severity ranked highest, followed by depression severity, TCI shyness, and baseline Y-BOCS compulsion and obsession scores (Fig. 4a). Across both outcomes, individual SHAP values were small in magnitude and showed considerable within-feature variability across patients, indicating that although these features are the most informative available, they do not drive predictions consistently enough for reliable individual-level classification. Notably, baseline Y-BOCS scores ranked among the top features for remission prediction but were ranked at the bottom for response prediction; a pattern consistent with the known sensitivity of the absolute remission threshold to baseline severity proximity.

When OCD-specific clinical variables were excluded, the feature importance rankings shifted partially, but notable consistencies remained (Supplementary Figs. 4 and 9). For response prediction, TCI fatigability and socioeconomic status retained their top positions across both clinical feature sets, suggesting these are among the more stable predictors independent of OCD-specific severity information. Depression severity (BDI-II) and trait anxiety (STAI-TRAIT) appeared consistently among the top-ranked features across both outcomes and both clinical feature sets, indicating that general psychopathology severity carries cross-cutting, if modest, predictive relevance. For remission prediction without OCD-specific variables, executive functioning emerged as the highest-ranked feature, a finding of potential clinical interest given the role of executive functioning in engagement with structured psychological therapies such as CBT.

For the multimodal response prediction, structural MRI features dominated the top 20 ranked features by mean absolute SHAP value (Supplementary Fig. 8). OCI-R checking severity was the only clinical variable in the upper portion of the ranking, with age of OCD onset appearing further down. This suggests that in the combined feature space, the model relies predominantly on structural brain information, though this did not translate into above-chance prediction performance. For the multimodal remission prediction (Fig. 4b), the two highest-ranked features were baseline Y-BOCS subscale scores, followed by a mixed set of clinical and structural MRI features, indicating that severity-related clinical information retains primacy even when neuroimaging data are available.

## Discussion

### Main discussion

In this study, we evaluated the prediction of treatment outcomes in OCD patients using a combination of demographic, clinical, and neuroimaging data through various ML models. We implemented a rigorous validation scheme to rule out data leakage, compared multiple ML algorithms to mitigate bias, and performed permutation testing to identify replicable treatment success markers. Treatment outcome was operationalized using two commonly employed binary labels: response, defined as a ≥ 35% reduction in Y-BOCS scores, and remission, defined as a post-treatment Y-BOCS score below the clinical threshold.

Despite extensive analyses in a well-characterized clinical sample, neither neuroimaging features alone nor multimodal models integrating neuroimaging with demographic and clinical variables produced prediction accuracies that exceeded chance level for either outcome criterion. Clinical and demographic features showed modest descriptive predictive value, particularly for remission prediction, where accuracies approached 66%, but these effects did not survive permutation testing. In contrast, prediction performance for response remained consistently at chance. Taken together, these findings suggest that robust individualized prediction of treatment outcome in OCD was not detectable in the present sample and modeling framework.

Although several clinical variables showed partial convergence with previous literature, no single feature or combination of features emerged as a consistently detectable predictors of treatment success. Prior ML studies similarly identified baseline OCD severity, depressive symptoms, and psychosocial functioning as modest but inconsistent predictors of remission^5,10–12^. Hilbert et al.^11^ achieved 65% balanced accuracy in predicting remission using baseline OCD severity, socioeconomic status, age, and depression scores, while Askland et al.^10^ achieved 75% balanced accuracy in a longitudinal sample, identifying baseline compulsion severity, social adjustment, and neuroticism as primary predictors. In the present study, SHAP-based feature importance rankings showed overlap with these findings. While we caution that these rankings are strictly descriptive and do not represent clinically meaningful predictors due to at-chance model performance, OCI-R symptom severity, depressive symptoms, socioeconomic status, and baseline Y-BOCS scores were among the most consistently ranked predictors across clinical models. Notably, baseline Y-BOCS severity appeared substantially more relevant for remission prediction than for response prediction.

A recognized fundamental challenge in extracting a consistent predictive signal is the clinical heterogeneity of OCD itself. As highlighted in a recent systematic review^49^, OCD is not a uniform disorder but a cluster of different phenotypes. Consequently, identical aggregate Y-BOCS scores may mask divergent symptom dimensions, etiologies, and cognitive profiles. This heterogeneity introduces substantial variance into the modeling process, making it significantly harder for supervised algorithms to identify generalizable patterns from baseline features.

Consistent with this complexity, the baseline clinical profiles of remitters and non-remitters showed differences across a range of measures. Specifically, non-remitters scored higher on measures of OCD symptom severity (washing, incompleteness), depressive symptoms, trait anxiety, neuroticism, shyness, anticipatory worry, and executive dysfunction. Rather than indicating distinct psychophenotypic subtypes, these differences are most consistent with a generalized severity and chronicity continuum in which patients with greater overall psychopathology and functional impairment are less likely to achieve favorable outcomes. For instance, elevated neuroticism and trait anxiety are well-established transdiagnostic markers of poorer therapeutic outcomes^50,51^. Furthermore, as highlighted in a recent review^49^, executive dysfunction may act as a specific barrier to the cognitive flexibility and active engagement required for successful CBT.

This severity dimension has direct implications for how remission-specific prediction results should be interpreted. Because remission is defined by an absolute post-treatment symptom threshold, patients entering treatment with lower baseline severity require a smaller absolute symptom reduction to achieve remission. Consequently, models assigning strong weight to baseline Y-BOCS scores may partly recover proximity to the remission threshold rather than individualized treatment prognosis. Remission prediction should therefore be interpreted cautiously as reflecting the likelihood of reaching a low-symptom endpoint rather than purely predicting treatment-related change. This interpretation is consistent with the observation that baseline severity contributed more strongly to remission prediction than to response prediction. In contrast, the response criterion is based on proportional symptom reduction and is therefore substantially less dependent on initial symptom severity.

Given the distinct limitations of each criterion when applied in isolation, we retained both response and remission as parallel outcome measures. The response criterion captures clinically meaningful proportional symptom improvement but may represent an overly stringent bar for patients entering with mild-to-moderate baseline severity, while the remission criterion reflects a clinically meaningful absolute symptom endpoint but is susceptible to the threshold proximity effects described above. Reporting both criteria in parallel partially offsets these complementary shortcomings and allows the degree of convergence between them to serve as an additional interpretive lens.

A counterintuitive pattern in the data illustrates this difference concretely: seven patients achieved remission without meeting the response criterion. This subgroup consists of patients who entered the study near the lower bound of the inclusion criterion, for whom a modest absolute improvement was sufficient to achieve remission but fell just short of the reduction threshold required for response. Rather than a data anomaly, this subgroup is a direct consequence of combining an absolute with a relative criterion.

Beyond the choice between outcome criteria, these considerations also bear on the decision to use binary rather than continuous outcomes. The practical goal is to identify patients at elevated risk of non-response so that augmented or alternative treatment pathways can be considered from the outset. A binary classification maps directly onto this decision, while a regression model’s output would still require a threshold to translate into an actionable clinical recommendation, a step that is no easier to calibrate and that does not circumvent the underlying classification problem. To fully address the potential inflation of baseline influence due to threshold dependency, we conducted a supplementary regression analysis predicting percent symptom change as a continuous outcome (see Supplementary Material). Despite being theoretically more powerful and less susceptible to threshold effects, this continuous approach likewise did not yield a detectable predictive signal. The consistency of findings across both binary and continuous operationalizations suggests that the lack of a predictive signal is unlikely to be explained solely by outcome dichotomization.

Turning to neuroimaging predictors, neither structural nor resting-state fMRI features yielded above-chance prediction, despite a systematic and progressive approach to feature representation. Previous studies examining fMRI network connectivity in OCD have not yet identified a coherent set of predictive features^19–22^. Therefore, we evaluated multiple levels of feature abstraction in sequence, including whole-brain functional connectivity matrices with regularization-based dimensionality control, PCA/ICA-based feature reduction, and compact global graph measures summarizing large-scale network topology. While necessary to manage our sample size, whole-brain PCA/ICA risks discarding weak localized signals. To ensure that dimensionality reduction did not obscure critical information, we conducted a supplementary hypothesis-driven analysis using unreduced amygdala–vmPFC connectivity^19^. This targeted approach also yielded chance-level predictions (see Supplementary Material), supporting the conclusion that the findings are not specific to PCA-based feature reduction. That above-chance prediction was not achieved at any level of feature abstraction may reflect the limited sample size available for imaging analyses as much as a genuine absence of relevant signal in the functional connectivity data. Node-level graph metrics were not included in the present analyses but represent a meaningful extension for future work, e.g. applied to frontolimbic cicuits^19^ or default-mode network nodes^21^. In line with a previous large-scale study^26^, structural MRI features similarly did not contribute to prediction.

The difficulty in establishing individualized brain-based predictions aligns with recent literature discussing the feasibility of finding associations between psychological phenotypes such as clinical presentation and imaging-derived markers. Large-scale studies^52,53^ suggest that sample sizes in the range of hundreds or thousands of patients might be needed for this task. Two recent treatment outcome prediction studies in mental disorders^54,55^ examined generalization performance of their ML models on independent clinical cohorts. Classification accuracies in the studies exceeded chance level but were rather low overall (58% in 55; 56% − 67%, in 54) and the models failed to generalize to external datasets. Ruffle and colleagues^56^ conducted a systematic evaluation of the predictability of individual characteristics from structural and fMRI data and reported poor prediction performance for psychological characteristics. In the majority of cases, models based on neuroimaging data did not contribute to prediction performance of participants’ mental health variables obtained from other health-related features.

A review on treatment outcome prediction in mental health interventions by Vieira et al.^9^ finds the field to be subject to publication bias with an over-reporting of larger effects. These stronger results stem from studies with small sample sizes and are not matched by studies demonstrating the same effects at larger scales. Supporting the conclusion by Vieira and colleagues^9^, several previous studies have employed less rigorous or no CV schemes and no permutation testing, increasing the risk of inflated effect sizes. This particularly afflicts neuroimaging studies that typically examine smaller sample sizes. In combination with overreporting of positive results this may have painted an overoptimistic picture of the prediction accuracies achievable for current ML models in precision psychotherapy.

In this light, our findings are consistent with the broader challenge of predicting psychological outcomes, potentially due in part to label noise^57^, even in relatively large samples. This challenge is likely to be even greater for neuroimaging studies, which generally require substantially larger sample sizes to reliably detect predictive signals.

### Limitations

Our dataset allowed us to perform CV; however, its relatively small sample size limited the power of our ML analyses (see Supplementary Analysis 3). For neuroimaging analyses, particularly fMRI, the reduced sample size may have been insufficient to achieve above-chance predictive performance. The dataset also lacked sufficient power to detect statistically significant effects in models descriptively performing above-chance. For example, although analyses including the established predictor symptom severity showed classification accuracies of up to 66% for remission, the sample size might not have provided adequate power to confirm these results statistically.

The neuroimaging analyses were subject to a particularly challenging dimensionality-to-sample-size ratio. Structural MRI yielded 496 features, and resting-state functional connectivity matrices span a substantially larger feature space prior to dimensionality reduction. Several methodological choices were made to mitigate this problem. First, we used nested CV to obtain unbiased performance estimates. For dimensionality reduction of functional connectivity features, we applied PCA/ICA. We further used regularized classifiers with penalization tuned in the inner loop, and variance thresholding to remove uninformative features prior to modelling. Finally, we used global graph summary measures rather than node-level metrics to keep the feature space compact. These steps reduce overfitting risk but cannot compensate for a fundamental limitation of statistical power. The null imaging results may therefore reflect the constraints of the current sample and design at least as much as a genuine absence of predictive signal. Replication in larger, adequately powered cohorts would help clarify the utility of neuroimaging features for treatment outcome prediction in OCD.

Better clinical prediction may be achieved by considering more fine-grained input data. Our clinical feature set included a variety of questionnaires assessing OCD symptomatology, comorbidities, and personality traits. However, we only used summed subscale scores from these instruments. Previous work by Askland et al.^10^ and Hilbert et al.^11^ suggests that individual items measuring OCD symptomatology may constitute predictors beyond overall symptom severity. Item-level features may therefore provide valuable additional information for prediction analyses. Future studies should exploit this source of information by focusing on deep phenotyping involving specific sets of relevant symptoms that are interrogated at a fine-grained level rather than a broad set of questionnaires covering many areas of impairment. With such fine-grained data, future research could also employ data-driven clustering approaches to empirically determine whether complex clinical profiles represent discrete etiological phenotypes or simply endpoints along a shared severity dimension.

Furthermore, our dataset did not include detailed characteristics of the therapeutic process itself. While our models were specifically designed to utilize pre-treatment data to inform decisions at the outset of therapy, CBT-related process variables, such as specific protocol components, therapeutic alliance ratings, homework adherence, therapist fidelity scores, and session-by-session symptom change trajectories, are critical factors in determining treatment success. Future studies that prospectively record these process variables alongside comprehensive baseline assessments are highly encouraged. Integrating these factors into ML frameworks could capture mechanisms of change that baseline assessments cannot, potentially yielding stronger prediction models than those achievable from pre-treatment data alone.

We acknowledge as a limitation our use of a last-observation-carried-forward approach for the 26 patients who discontinued therapy prematurely. While this strategy can potentially bias outcomes, our data suggests that premature discontinuation did not systematically correspond to poor treatment outcome. Relatedly, the substantial heterogeneity in treatment dose and duration across our sample may serve as a source of label noise. However, our supplementary analyses offer reassurance that session count itself does not carry a strong, systematic predictive signal.

Finally, we compared the outcome labels response and remission, which are subject to considerable label noise since they were based on Y-BOCS scores alone. Additional related clinical or real-life measurements may improve the quality of those labels. For example, quality of life measures, social functioning, or outcome measures that are based on longer time intervals may potentially be more robust, as seen in Askland et al.^10^ who used longitudinal measures assessing longer time ranges to determine the criterion (percent time spent in remission and ever experiencing remission that lasted at least 8 weeks). Using such a criterion, treatment outcome was predictable with 75% accuracy. Future studies with larger samples should explore continuous outcome modeling as a complementary approach, as such analyses would be free of the threshold dependency introduced by binary remission criteria.

### Conclusion

In this study, we used a combination of demographic, clinical, and neuroimaging data to predict treatment outcome in OCD patients following CBT. While clinical predictors descriptively performed better than neuroimaging features, no statistically significant predictive signal was detectable in the present sample, and integrating neuroimaging data did not enhance prediction accuracy beyond demographic and clinical variables alone. These findings align with broader literature highlighting the limitations of current neuroimaging techniques for individualized prediction in mental health but need to be cautiously interpreted given their feature dimensionality relative to the sample size. The modest prediction accuracies underscore the need for larger samples, as well as more fine-grained input features and more reliable outcome criteria. Future research should focus on deep phenotyping and longitudinal studies to enhance the robustness and applicability of predictive models.

## Supplementary Information

Below is the link to the electronic supplementary material.

Supplementary Material 1

## Data Availability

Data is not available to be shared due to data protection agreements. All code used for data analysis is provided under https://github.com/ritterlab/EPOC_outcome_prediction.git.

## Declarations

### **Generative AI and AI-assisted technologies in the writing process**

During the preparation of this work the author(s) used GPT-4o in order to improve readability and language. After using this tool/service, the author(s) reviewed and edited the content as needed and take(s) full responsibility for the content of the publication.
