# Supplementary material for: Evaluating clinical and neuroimaging predictors for cognitive-behavioral therapy outcome in obsessive-compulsive disorder
Source: Sci Rep. 2026 Aug 8;16:24542. doi: 10.1038/s41598-026-64405-y (PMC13452815; doi:10.1038/s41598-026-64405-y)
Supplement: Supplementary file 1 [file 41598_2026_64405_MOESM1_ESM.pdf]

Supplement

Evaluating clinical and neuroimaging predictors for cognitive-behavioral therapy outcome in obsessive-compulsive disorder

Marija Tochadse[1,2,3], Julia Klawohn[3,4], Christian Kaufmann[3], Rosa Grützmann[3,5], Anja Riesel[3,6], Stephan Heinzel[3,7], Roshan Prakash Rane[1,2,3], Sofia Pereira da Silva[8,9], Sarah Wellan[1], Sam Gijsen[1,2], Kerstin Ritter[1,2,8], Norbert Kathmann[3]

Supplementary Table 1. Value ranges used for hyperparameters tuning.

Supplementary Table 2. Mean cross-validated balanced accuracy (BA), 95% confidence intervals (based on the standard error of the mean across outer folds), and exact permutation p-values for all models predicting treatment response and remission

Supplementary Analysis 1. Predicting Continuous Symptom Change

Supplementary Analysis 2. Targeted Hypothesis-Driven Connectivity Features

Supplementary Analysis 3. Statistical Power and Sensitivity Analysis via Simulation

Supplementary Figure 1-3. Simulation-based sensitivity analyses

Supplementary Figure 4-11. SHAP-based features importances for response and remission models

Model	Hyperparameters	Values tested
Logistic regression	C: Inverse of L2 regularization strength	10000, 1000, 100, 1.0, 0.001, 0.0001
Linear SVM	C: Inverse of L2 regularization strength	10000, 1000, 100, 1.0, 0.001, 0.0001
Kernel-based SVM	C: Inverse of L2 regularization strength,<br>γ: kernel coefficient of RBF kernel	10000, 1000, 100, 1.0, 0.001, 0.0001<br>'auto', 'scale'
Gradient boosting	Learning rate,<br>Maximum depth,<br>Number of estimators	0.01, 0.1, 0.25<br>3, 5<br>50, 100, 200

Supplementary Table 1. Value ranges used for hyperparameters tuning.

Analysis	Model	Response			Remission		
		Mean CV BA	95% CI (SEM)	*p*-value (uncorrected)	Mean CV BA	95% CI (SEM)	*p*-value (uncorrected)
**Tabular – OCD**	GB	0.467	[0.4466, 0.4868]	–	0.661	[0.6365, 0.6859]	.21
	LR(c)	0.481	[0.4663, 0.4964]	–	0.644	[0.6163, 0.6711]	.28
	SVM-lin	0.496	[0.4730, 0.5194]	–	0.650	[0.6204, 0.6793]	.28
	SVM-rbf	0.472	[0.4530, 0.4901]	–	0.650	[0.6261, 0.6748]	.25
**Tabular**	GB	0.468	[0.4442, 0.4916]	–	0.623	[0.5947, 0.6507]	.30
	LR(c)	0.474	[0.4582, 0.4893]	–	0.607	[0.5793, 0.6355]	.39
	SVM-lin	0.491	[0.4721, 0.5099]	–	0.630	[0.6017, 0.6577]	.27
	SVM-rbf	0.475	[0.4566, 0.4935]	–	0.587	[0.5616, 0.6118]	.45
**sMRI**	GB	0.503	[0.4812, 0.5246]	–	0.519	[0.4911, 0.5463]	–
	LR(c)	0.510	[0.4863, 0.5346]	–	0.538	[0.5061, 0.5709]	–
	SVM-lin	0.494	[0.4731, 0.5150]	–	0.536	[0.5088, 0.5630]	–
	SVM-rbf	0.571	[0.5469, 0.5942]	.39	0.537	[0.5169, 0.5562]	–
**fMRI**	GB	0.476	[0.4542, 0.4974]	–	0.501	[0.4697, 0.5317]	–
	LR(c)	0.500	[0.4648, 0.5348]	–	0.538	[0.5074, 0.5685]	–
	SVM-lin	0.534	[0.5001, 0.5682]	–	0.564	[0.5291, 0.5999]	*
	SVM-rbf	0.508	[0.4942, 0.5215]	–	0.538	[0.5119, 0.5644]	–
**fMRI – PCA**	GB	0.502	[0.4803, 0.5242]	–	0.509	[0.4789, 0.5392]	–
	LR(c)	0.500	[0.4711, 0.5284]	–	0.532	[0.4968, 0.5679]	–
	SVM-lin	0.443	[0.4145, 0.4718]	–	0.512	[0.4791, 0.5455]	–
	SVM-rbf	0.500	[0.4709, 0.5291]	–	0.538	[0.5104, 0.5664]	–
**fMRI – ICA**	GB	0.519	[0.4931, 0.5454]	–	0.505	[0.4740, 0.5355]	–
	LR(c)	0.493	[0.4709, 0.5155]	–	0.522	[0.4898, 0.5537]	–
	SVM-lin	0.485	[0.4649, 0.5059]	–	0.533	[0.4991, 0.5661]	–
	SVM-rbf	0.495	[0.4797, 0.5094]	–	0.522	[0.4879, 0.5556]	–
**fMRI – graph**	GB	0.561	[0.5311, 0.5916]	.42	0.446	[0.4140, 0.4789]	–
	LR(c)	0.485	[0.4684, 0.5010]	–	0.453	[0.4259, 0.4808]	–
	SVM-lin	0.506	[0.4821, 0.5306]	–	0.444	[0.4108, 0.4766]	–
	SVM-rbf	0.453	[0.4275, 0.4778]	–	0.441	[0.4057, 0.4769]	–
**Multimodal**	GB	0.535	[0.5112, 0.5597]	–	0.559	[0.5276, 0.5910]	.51
	LR(c)	0.494	[0.4686, 0.5202]	–	0.595	[0.5604, 0.6290]	.28
	SVM-lin	0.508	[0.4827, 0.5342]	–	0.581	[0.5465, 0.6163]	.40
	SVM-rbf	0.548	[0.5266, 0.5691]	–	0.551	[0.5333, 0.5691]	.58
**Amy-vmPFC**	GB	0.512	[0.4813, 0.5429]	–	0.497	[0.4601, 0.5346]	–
	LR(c)	0.489	[0.4798, 0.4987]	–	0.463	[0.4320, 0.4946]	–
	SVM-lin	0.503	[0.4707, 0.5343]	–	0.492	[0.4574, 0.5266]	–
	SVM-rbf	0.464	[0.4388, 0.4898]	–	0.471	[0.4350, 0.5070]	–

Supplementary Table 2. Mean cross-validated balanced accuracy (BA), 95% confidence intervals (based on the standard error of the mean across outer folds), and exact permutation p-values for all models predicting treatment response and remission. p-values are not corrected for multiple comparisons reported only for models with balanced accuracy > 0.55; "–" indicates balanced accuracy below this threshold and p-value not computed, “*” indicates the exact permutation p-value not computed for this model due to the computational cost associated with the large number of input features.

Supplementary Analysis 1. Predicting Continuous Symptom Change

Rationale: To determine whether the limited predictive performance observed in our binary classification tasks (response and remission) was an artifact of dichotomizing the outcome variables, we conducted a supplementary regression analysis. Because the absolute threshold defining remission (Y-BOCS < 12) is sensitive to baseline severity, predicting a continuous outcome (percent symptom change) offers a less threshold-dependent and potentially statistically more powerful approach to modeling treatment-related improvement.

Methods: We utilized the same clinical input features, including OCD-specific variables, to predict the percent change in Y-BOCS scores from pre- to post-treatment. We implemented a regression framework entirely analogous to our primary classification pipeline, training four distinct algorithms: Ridge Regression, Linear Support Vector Regression (SVR), SVR with a radial-basis function (RBF) kernel, and XGBoost regression. The hyperparameter tuning, cross-validation strategy, and evaluation procedures were identical to those employed in the main classification framework.

Results: Consistent with our primary classification results, the regression analysis yielded no predictive signal. Evaluation on the held-out data resulted in R^2 values ranging between -0.07 and -0.09 across all four models. These findings indicate that the continuous models failed to outperform a simple mean-prediction baseline. Consequently, these results reinforce our primary conclusions, confirming that the lack of predictive success is likely driven by the limited prognostic information contained within the available clinical features rather than the specific threshold-based operationalization of the clinical outcome.

Supplementary Analysis 2. Targeted Hypothesis-Driven Connectivity Features

Rationale: While dimensionality reduction via PCA was a necessary step to manage the high-dimensional whole-brain connectivity matrix relative to our sample size (n=80), this approach inherently risks discarding weak but potentially meaningful signals localized to specific neural circuits. To address whether a more targeted, hypothesis-driven approach could uncover predictive utility without the need for PCA, we conducted a supplementary analysis focusing exclusively on the functional connectivity between the amygdala and the ventromedial prefrontal cortex (vmPFC) - a core circuit highlighted in literature regarding and OCD pathology (Fullana et al., 2017).

Methods: We extracted the specific, unreduced functional connectivity values between the amygdala (lAmyg left and right) and vmPFC (A11l left and right) from the brainnetome atlas used to obtain our connectivity matrices. These targeted, hypothesis-driven features were then input into our established machine learning pipeline to predict treatment response and remission. All modeling steps, including the algorithms utilized (Ridge, Linear SVR, RBF SVR, XGBoost), hyperparameter tuning, and cross-validation strategies, were identical to the primary analysis.

Results: The targeted amygdala-vmPFC models failed to exceed chance-level performance. For predicting treatment response, the models achieved balanced accuracies ranging between 46% and 51%. Similarly, for predicting remission, balanced accuracies ranged between 46% and 50%. These findings provide no evidence that this specific, hypothesis-driven neural circuit holds predictive prognostic information for treatment outcomes in our current sample. This aligns with our primary, data-driven PCA findings, corroborating the conclusion that the lack of robust predictive signal is likely not an artifact of broad dimensionality reduction.

Supplementary Analysis 3. Statistical Power and Sensitivity Analysis via Simulation

1. Objective

To robustly interpret the statistical significance of our ML predictions and to contextualize the observed Balanced Accuracies (BA) given our specific sample size, we conducted a comprehensive simulation-based power and sensitivity analysis. The primary goal of this simulation was to determine the Minimum Detectable Effect (MDE) of our exact ML pipeline and to systematically evaluate how different permutation-based significance testing frameworks impact statistical power.

2. Feature Standardization and Latent Signal Construction

To ensure the simulation was grounded in the true mathematical properties of our empirical dataset, the original input feature matrices (X) for both the tabular clinical/demographic and multimodal data were retained. A synthetic latent signal (z) was then constructed by taking a linear combination of the empirical features. Specifically, a coefficient vector (v) was drawn from a standard normal distribution. To simulate a scenario where all features contribute to the outcome, all coefficients were non-zero. The raw continuous signal was computed as $z = Xv$, which was subsequently standardized to have zero mean and unit variance.

3. Synthetic Label Assignment and Class Balance Preservation

To translate the continuous latent signal (z) into binary classification targets (y) while perfectly maintaining the empirical class balance, we utilized a parameterized logistic probability function. A signal strength parameter (beta) was used to scale the latent signal before passing it through an expit (logistic sigmoid) function: $p = \text{expit}(\beta * z)$. The parameter beta effectively dictates the signal-to-noise ratio.

To preserve the exact original class distribution, we assigned exactly m positive labels (where m is the true number of positive cases in the empirical dataset). The indices for these m positive labels were drawn without replacement from the dataset, weighted by the individual logistic probabilities (p). When beta = 0, all probabilities are equal, effectively shuffling the targets completely randomly (representing the null hypothesis). As beta increases, the probability of assigning a positive label becomes increasingly dependent on the empirical feature combination, creating a true, predictable signal.

4. Oracle Balanced Accuracy (Theoretical Predictability)

For every synthetic dataset we calculated an oracle balanced accuracy: the balanced accuracy obtained by applying the best threshold to the latent signal z used to generate the labels. Thresholding z is the optimal decision rule, so this value reflects the separability of the planted signal given knowledge of the true signal direction. It serves as an interpretable, effect-size measure of signal strength, letting us report detectability on a balanced-accuracy scale rather than in units of the abstract signal parameter beta. Importantly, it is not the accuracy achievable from the features X. The pipeline never observes z and must recover the signal direction from X alone.

5. Machine Learning Pipeline Execution

For each constructed synthetic dataset, we executed the exact same cross-validated machine learning pipeline utilized in the main study. We recorded the out-of-sample BA for each run to verify that the empirical performance of the pipeline scaled appropriately with the theoretical predictability of the ground-truth synthetic targets.

6. Significance Testing Frameworks and Power Estimation

As there is no standard regarding the optimal calculation of permutation-based p-values in cross-validation settings, we evaluated the statistical power of the pipeline using two distinct significance testing frameworks. To establish empirical null distributions, we executed the complete cross-validation pipeline 1,000 times under the null condition (beta = 0. To evaluate statistical power, we iterated through varying signal strengths (beta in [0.5, 1.0, 1.5, 2.0, 3.0, 4.0, 6.0]), executing the full ML pipeline 100 times for each level.

Fold-Level Evaluation (Conservative): In this framework, the null distribution consisted of 70,000 individual Balanced Accuracies (BAs), generated from 7 outer folds across 10 repetitions for all 1,000 null runs. For each of the 100 runs generated at a specific signal strength (beta > 0), we extracted the 70 empirical BA values. For each individual empirical BA, a fold-level p-value was calculated by determining the proportion of the 70,000 null BAs that exceeded it. These 70 p-values were then averaged to compute a final, aggregate p-value for that specific run, with the run defined as "significant" if the average p-value was less than 0.05. Because evaluating performance on small test-fold subsets introduces high statistical variance, averaging these rank-based p-values proved to be highly conservative. This framework possessed adequate statistical power to detect only medium-to-strong effects in the tabular data, and even strong effects lacked statistical power in the high-dimensional neuroimaging data. It was severely underpowered to detect small effects.

Run-Level Aggregated Evaluation (More Liberal): To conduct the aggregated permutation test, we established the empirical null distribution by first averaging the fold-wise BAs within each pipeline run, resulting in a stabilized null distribution of exactly 1,000 averaged values. The 95th percentile of this aggregated distribution established our critical significance threshold. For the signal conditions, power was defined as the percentage of the 100 runs whose internally averaged BA exceeded this 95th percentile threshold. Benefitting from the massive stabilization of variance achieved by averaging across folds prior to significance testing, this framework demonstrated high statistical power for both medium and large effects, and maintained superior sensitivity for detecting small effects in the tabular data.

7. Limitations and Caveats

While this simulation provides a rigorous mathematical boundary for the statistical power of our study, we note several inherent limitations. Synthetic signal generation relies on predefined mathematical assumptions regarding the relationship between the features and the target (e.g., linearity, feature sparsity, and noise distribution). Because the true functional form of the biological and clinical relationships in our empirical data is unknown, the simulation cannot perfectly mirror real-world

complexity. Therefore, these power estimations should be interpreted carefully as theoretically sound approximations rather than absolute clinical certainties.

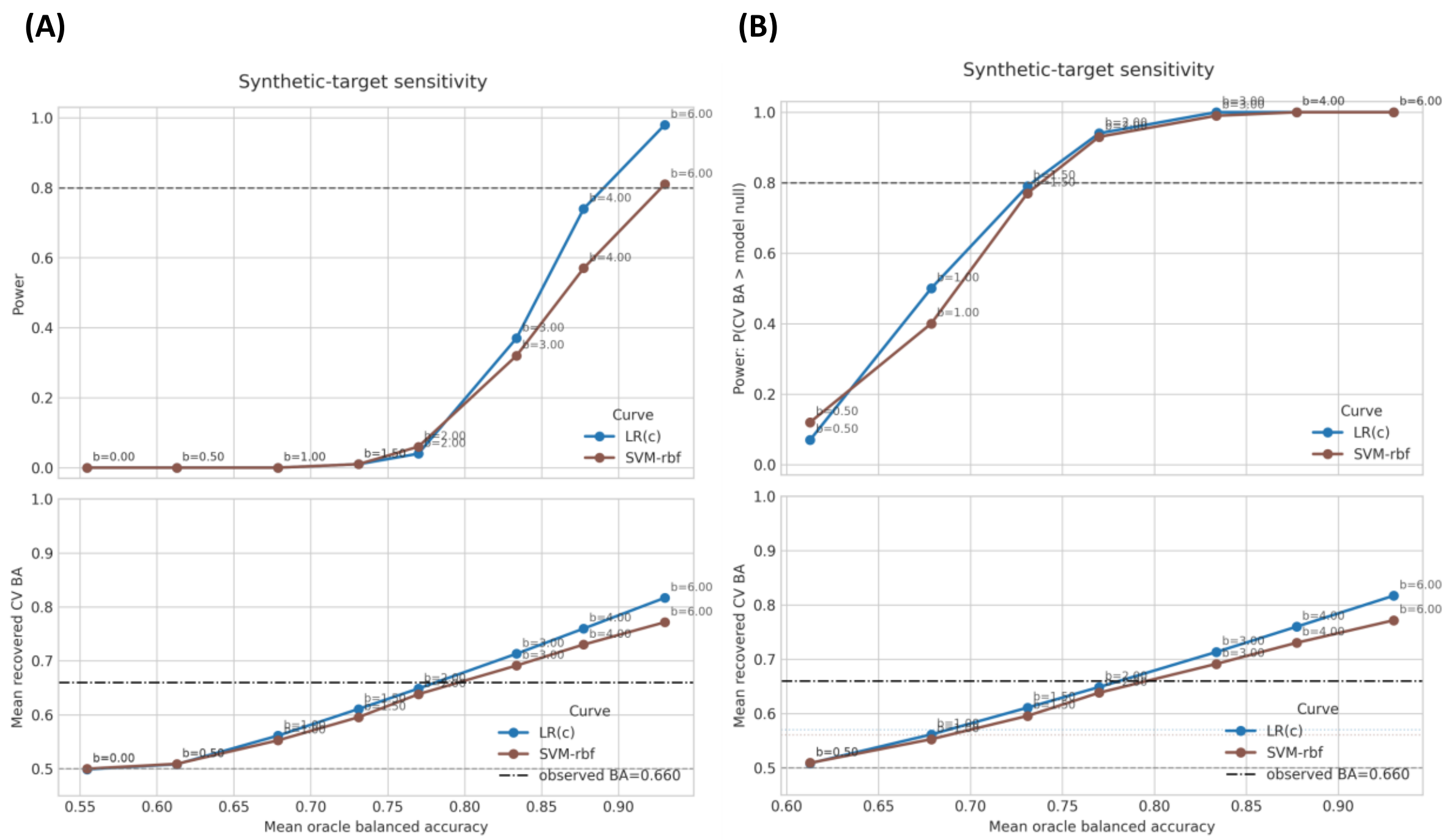

Supplementary Figure 1A, B. Simulation-based statistical power of the machine learning pipeline across increasing effect sizes for demographic and clinical input data (119 patients, 31 features). A fold-wise, **B** run-wise

(A, B) Top plots: This panel illustrates the statistical power of the machine learning models (y-axis) as a function of the theoretical predictability of the underlying synthetic signal, expressed as the Mean Oracle Balanced Accuracy (x-axis). Power is defined as the proportion of simulation runs at a given signal strength (beta) where the pipeline's Balanced Accuracy exceeded the null threshold. The horizontal dashed line marks the standard target threshold for adequate statistical power (80%). Annotated data points indicate the specific underlying signal strength (beta) parameter used to generate the synthetic target for that iteration.
(A, B) Bottom plots: This panel demonstrates the pipeline's capacity to capture the underlying biological signal by plotting the empirically achieved Mean Cross-Validated Balanced Accuracy (y-axis) against the theoretical maximum limit (Mean Oracle Balanced Accuracy, x-axis). The dashed horizontal line at 0.5 represents chance-level prediction. The dash-dotted black line indicates the actual empirical Balanced Accuracy observed in the primary study, allowing for a visual estimation of the theoretical predictability and statistical power corresponding to the study's real-world results.

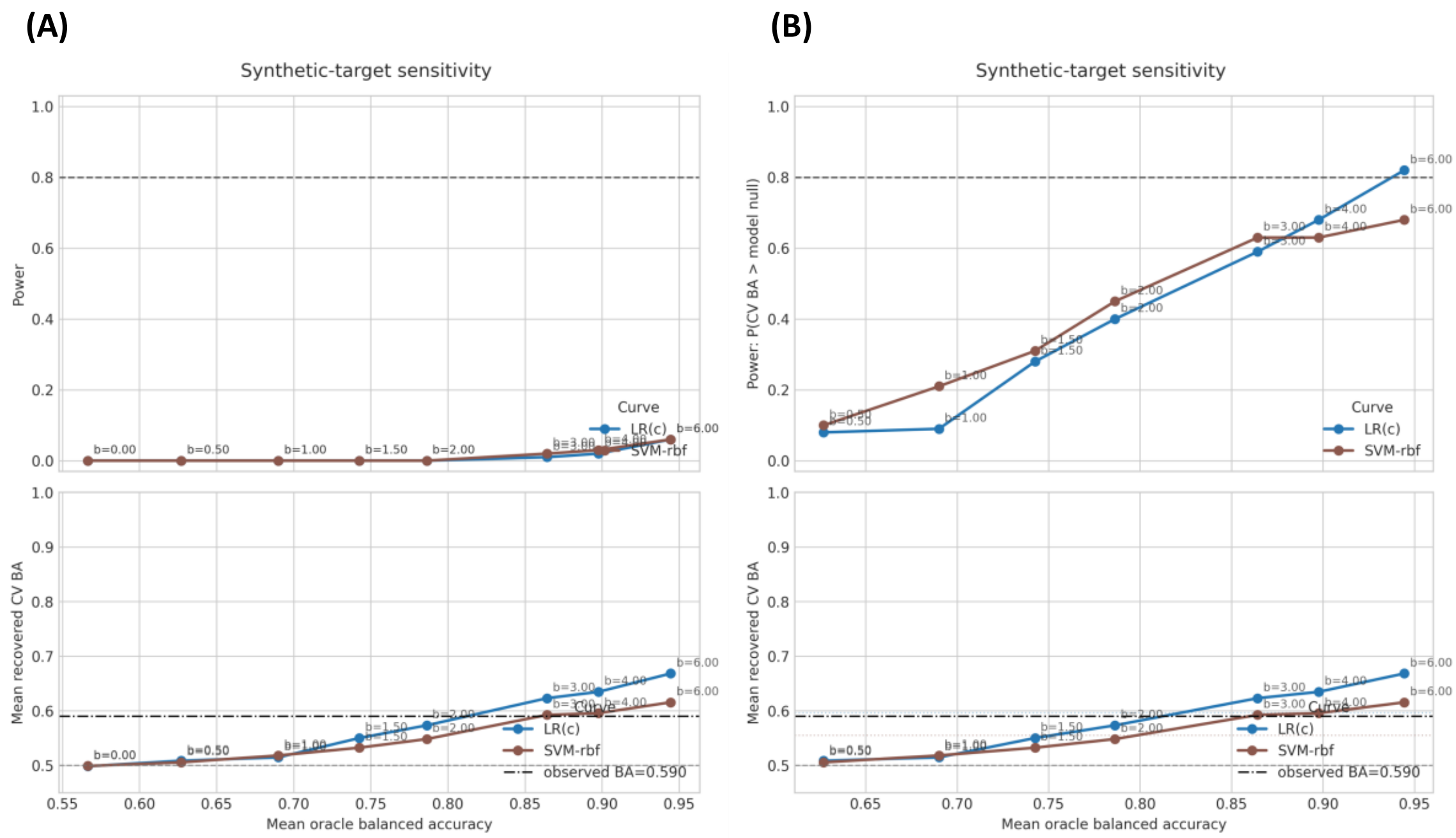

Supplementary Figure 2A, B. Simulation-based statistical power of the machine learning pipeline across increasing effect sizes for multimodal input data (80 patients, 500+ features). A fold-wise, **B** run-wise

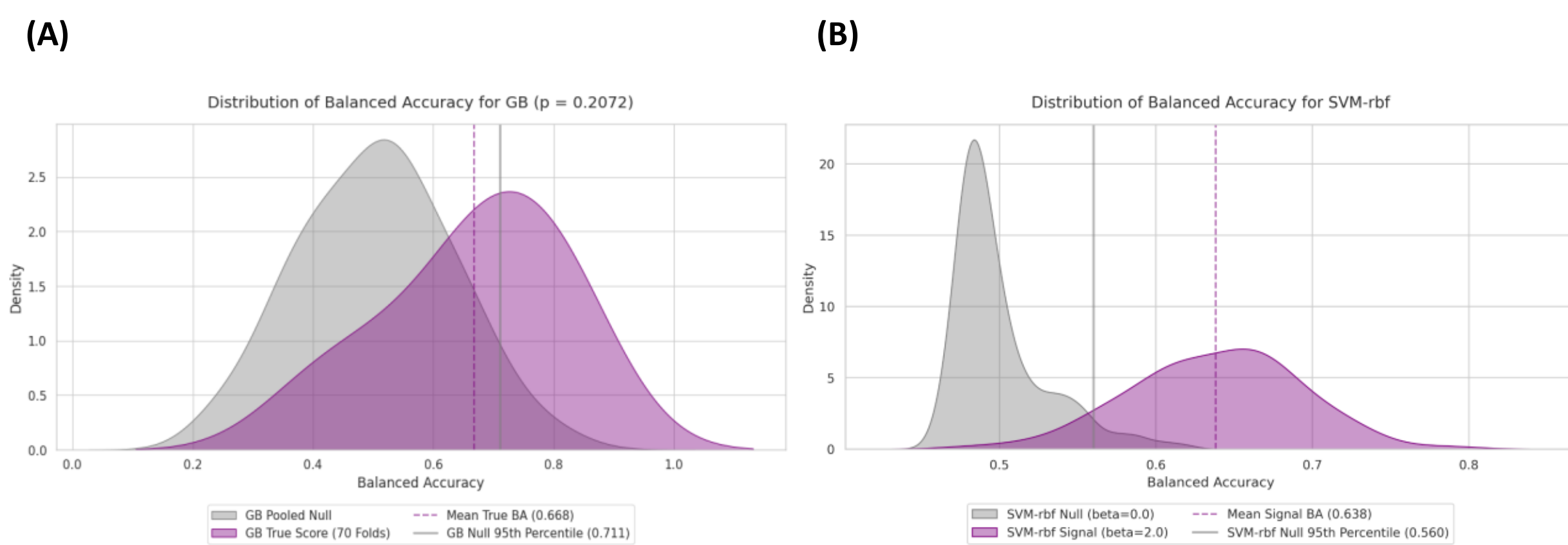

Supplementary Figure 3A, B. Comparison of null and empirical balanced-accuracy distributions based on demographic and clinical input data. (A) Fold-wise balanced accuracies for top-performing clinical model (GB), **B** Run-wise balanced accuracies for SVM-rbf model simulation

(**A**) This figure visualizes the empirical performance of the machine learning pipeline, explicitly comparing the variance of the true cross-validated model scores against the empirical null distributions. The gray curve represents the null distribution generated across the cross-validation repetitions, computed via random permutation of the target labels. The solid gray vertical line denotes the global 95th-percentile significance threshold, derived from pooling the permutation scores across all folds and repetitions.

The shaded purple area illustrates the distribution of the true, unpermuted Balanced Accuracies achieved by the model across all 70 individual cross-validation folds (i.e., 10 repetitions * 7 folds), demonstrating the inherent variance introduced by data splitting. The dashed purple line marks the model's overall mean true balanced accuracy. The final reported p-value (indicated in the title) reflects the highly conservative testing framework of calculating individual fold-level p-values against their respective repetition-level null distributions and subsequently averaging them. Comparing the mean true accuracy (dashed purple line) against the global 95th-percentile threshold (solid gray line) provides a visual approximation of the model's aggregate predictive signal relative to chance.

(B) This figure illustrates the kernel density estimates of the cross-validated Balanced Accuracy scores achieved by the machine learning pipeline under both null and simulated signal conditions.

The gray distribution (representing the Null Hypothesis, beta = 0.0) visualizes the empirical performance of the model when predicting synthetic targets that have no mathematical relationship to the input features. The gray vertical line marks the critical 95th-percentile significance threshold derived from this null distribution.

The purple distribution (representing a True Signal, beta = 2.0) illustrates the recovered empirical Balanced Accuracies when a synthetic, predictable signal is actively embedded in the target variable. The dashed purple line denotes the mean Balanced Accuracy of these signal runs. Comparing the overlap between these two distributions visually demonstrates the statistical power of the pipeline and how effectively a true underlying signal shifts the distribution of model predictions beyond the critical significance threshold.

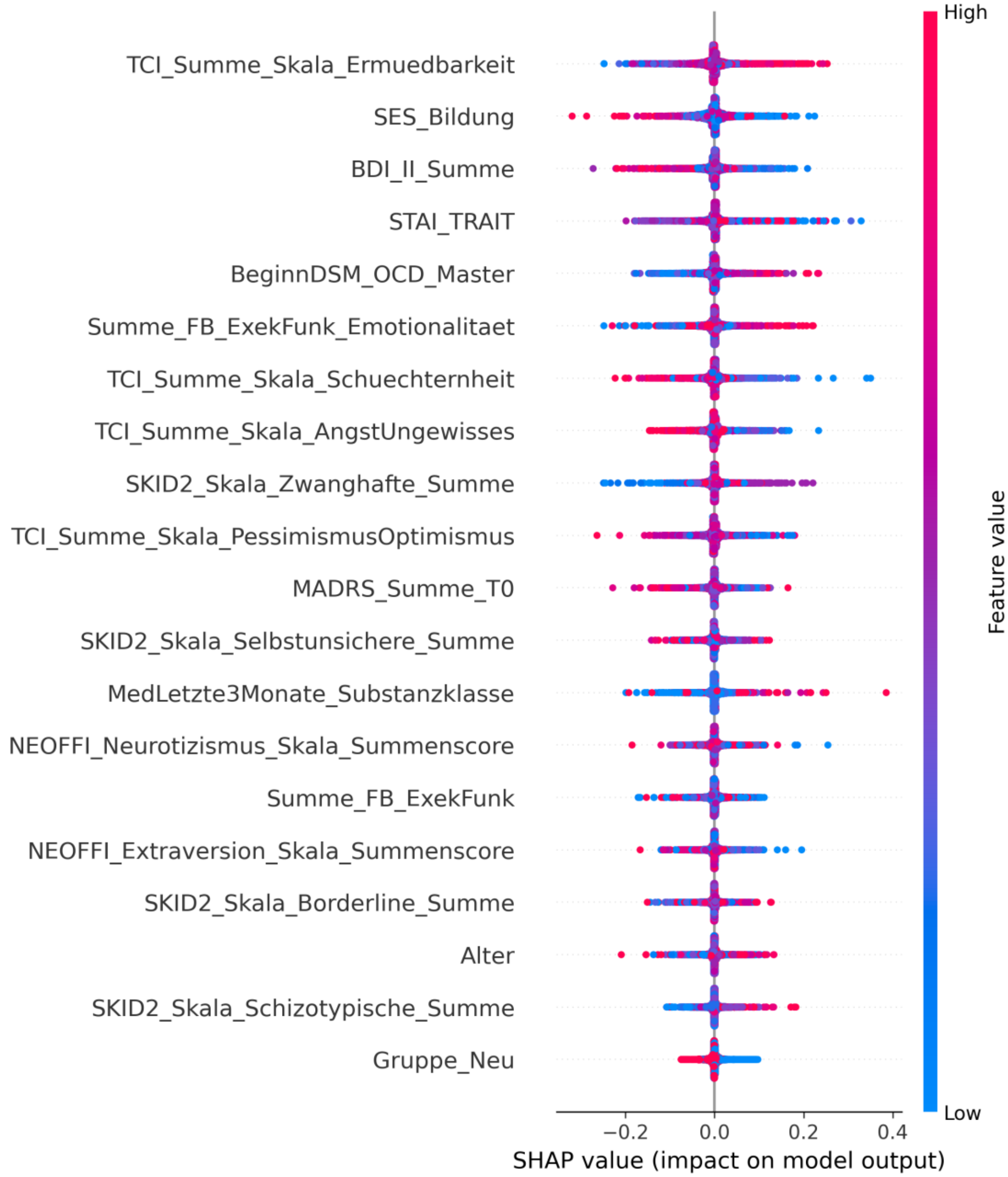

Supplementary Figure 4. Most informative Tabular clinical features (excluding OCD variables) for response prediction.

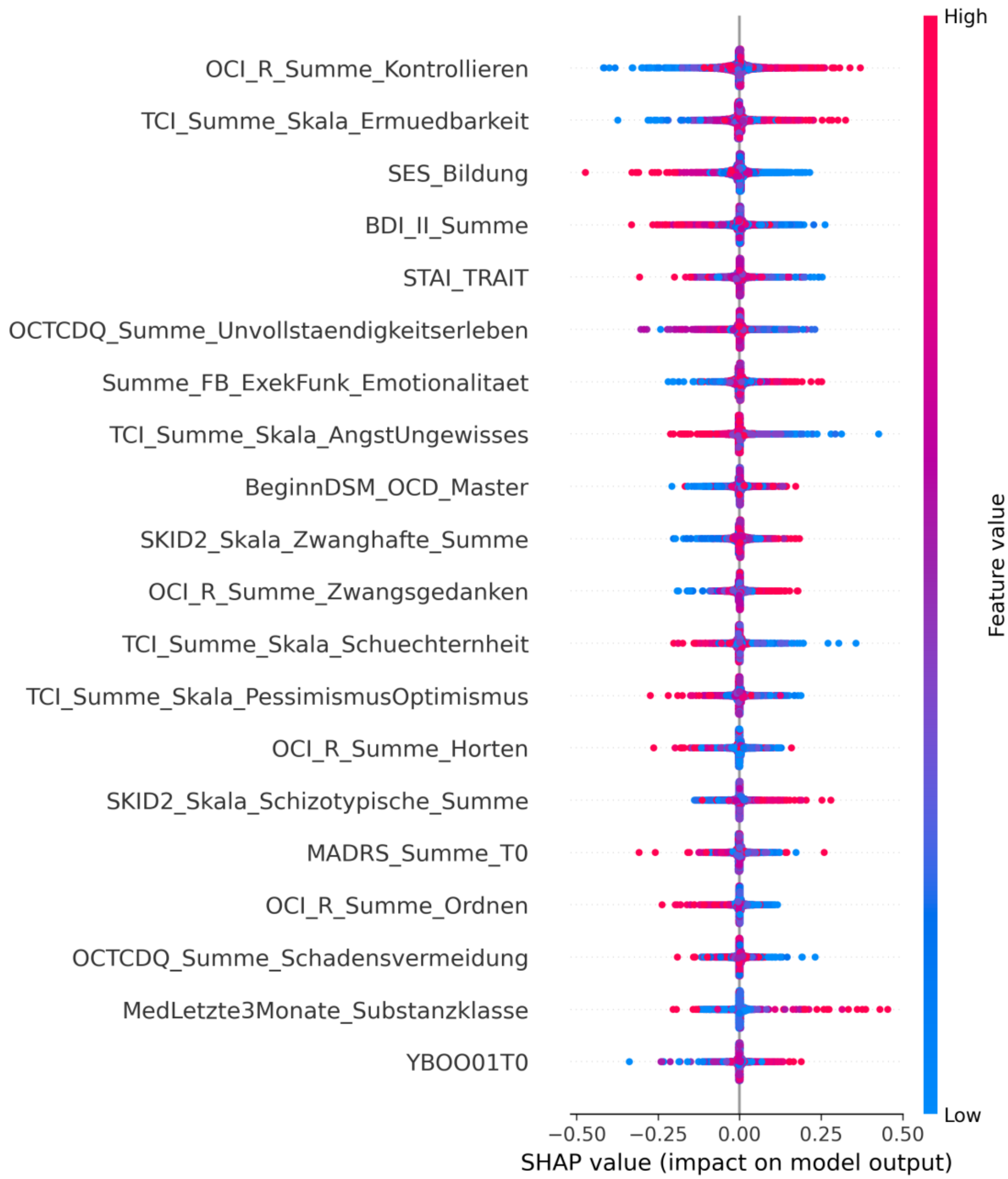

Supplementary Figure 5. Most informative Tabular clinical features (including OCD variables) for response prediction.

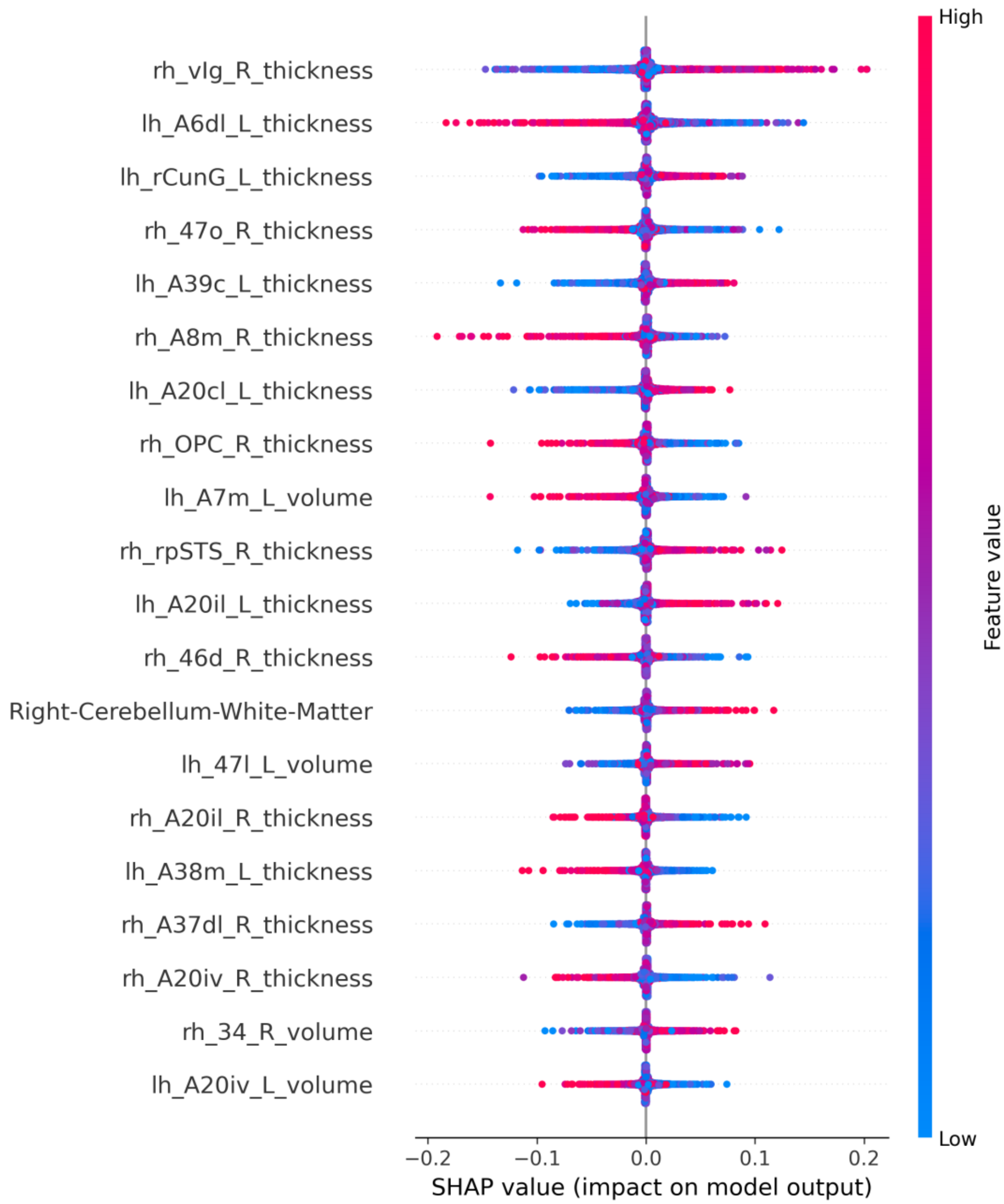

Supplementary Figure 6. Most informative structural MRI features for response prediction.

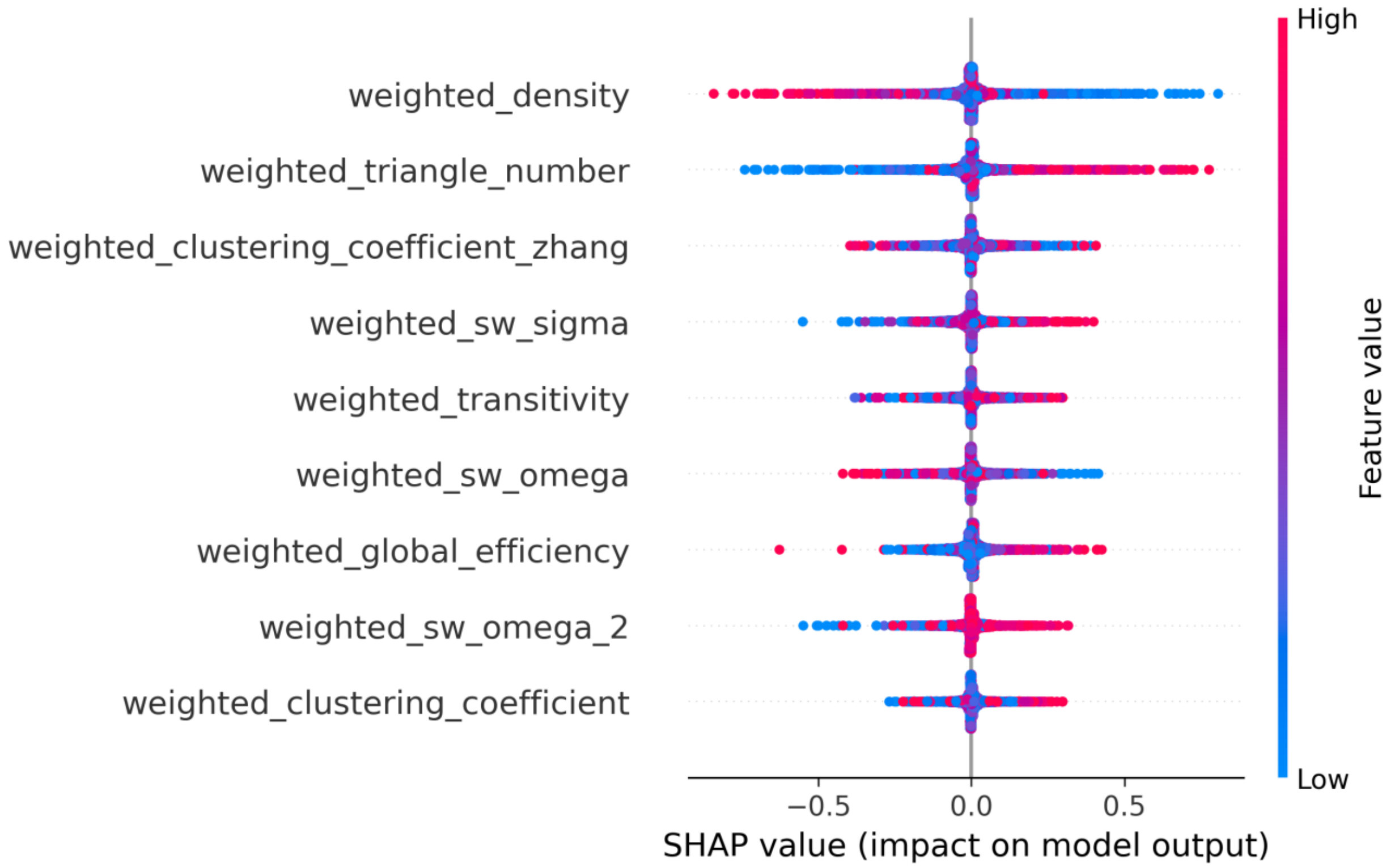

Supplementary Figure 7. Most informative global graph fMRI features for response prediction.

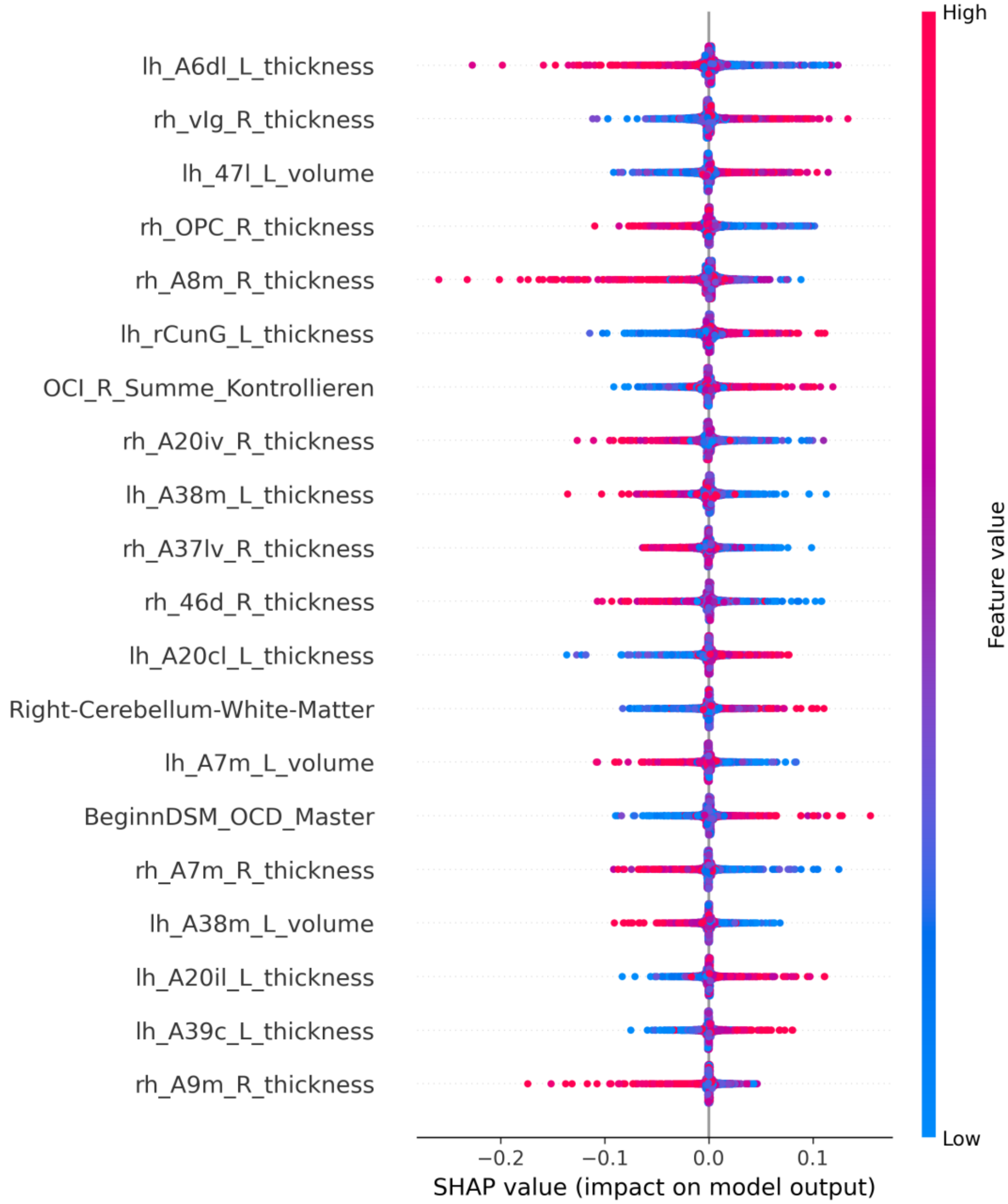

Supplementary Figure 8. Most informative features for multimodal response prediction.

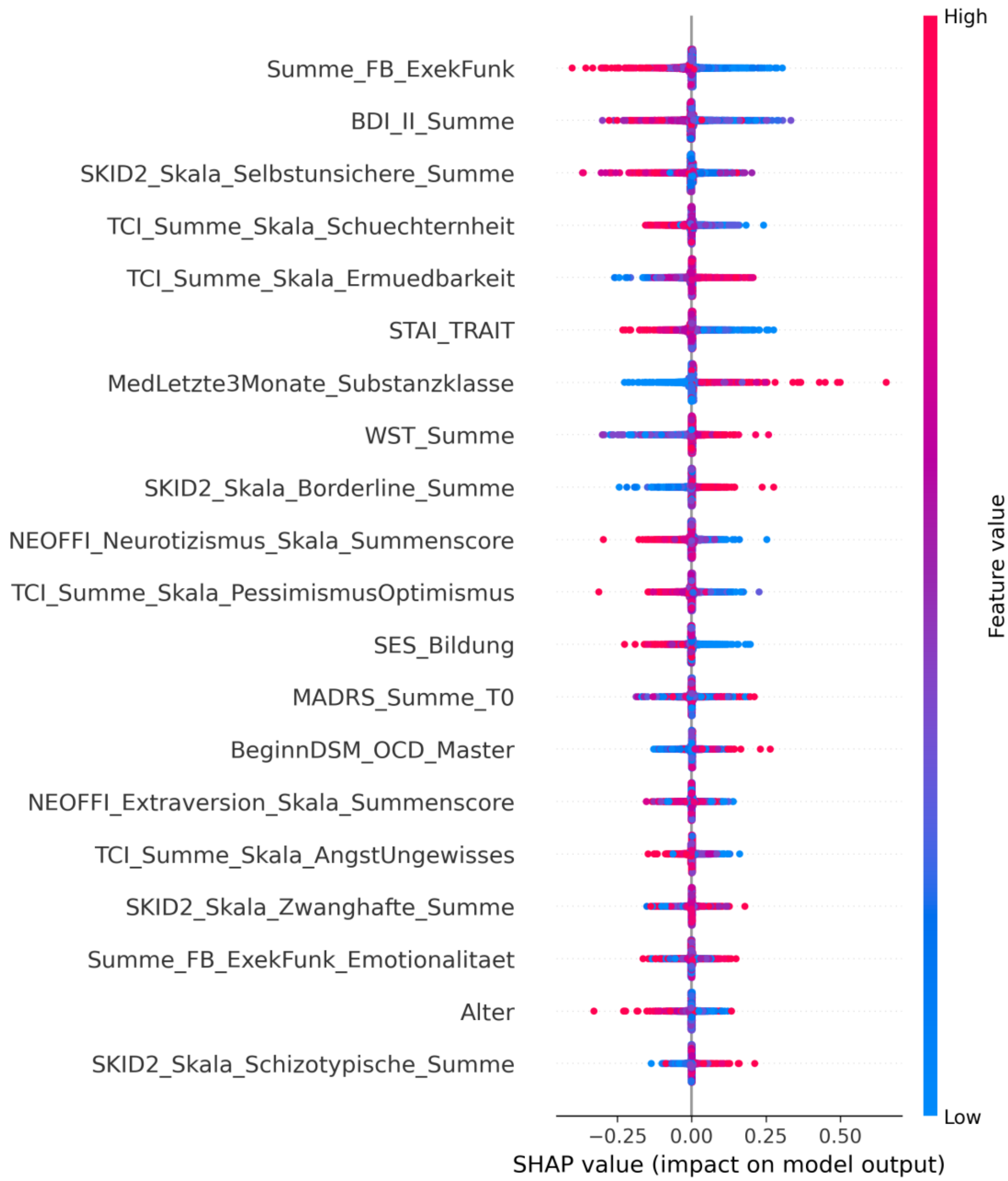

Supplementary Figure 9. Most informative Tabular clinical features (excluding OCD variables) for remission prediction.

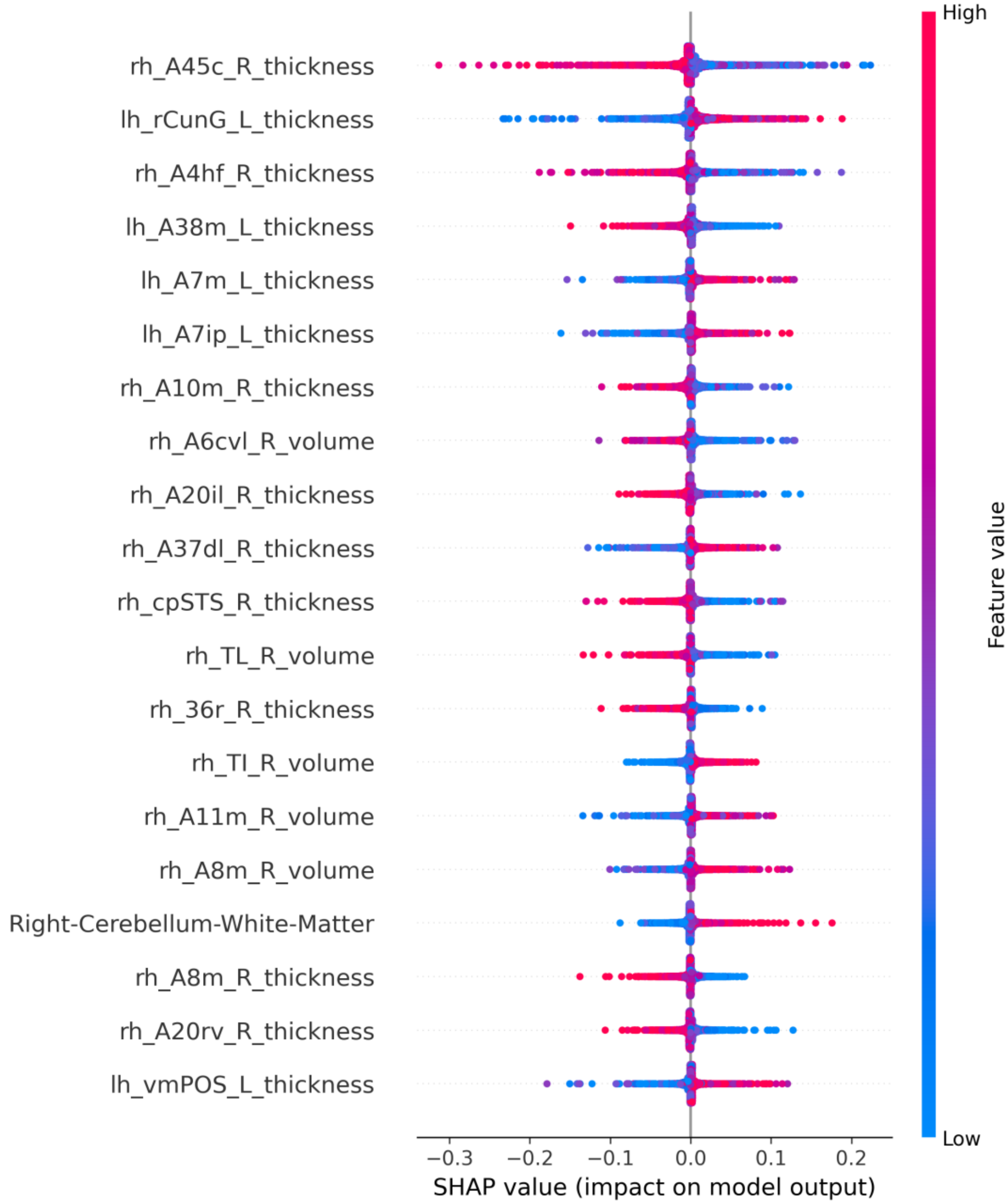

Supplementary Figure 10. Most informative structural MRI features for remission prediction.

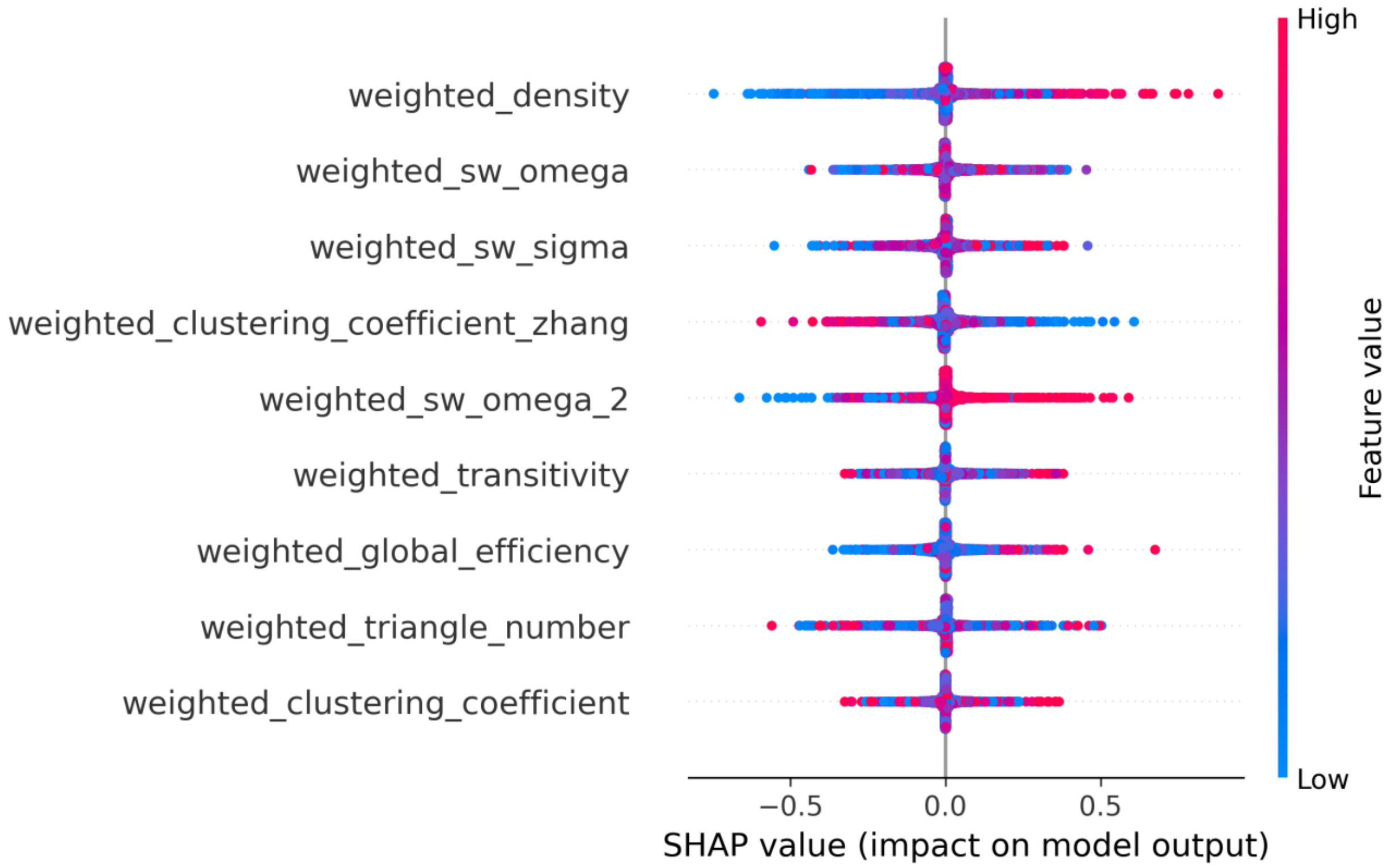

Supplementary Figure 11. Most informative global graph fMRI features for remission prediction.
